# Matriptase-2 deficiency protects from obesity by modulating iron homeostasis

**DOI:** 10.1038/s41467-018-03853-1

**Published:** 2018-04-10

**Authors:** Alicia R. Folgueras, Sandra Freitas-Rodríguez, Andrew J. Ramsay, Cecilia Garabaya, Francisco Rodríguez, Gloria Velasco, Carlos López-Otín

**Affiliations:** Departamento de Bioquímica y Biología Molecular, Facultad de Medicina, Instituto Universitario de Oncología del Principado de Asturias (IUOPA), Universidad de Oviedo, 33006 Oviedo, Spain

## Abstract

Alterations in iron status have frequently been associated with obesity and other metabolic disorders. The hormone hepcidin stands out as a key regulator in the maintenance of iron homeostasis by controlling the main iron exporter, ferroportin. Here we demonstrate that the deficiency in the hepcidin repressor matriptase-2 (Tmprss6) protects from high-fat diet-induced obesity. *Tmprss6*^*−/−*^ mice show a significant decrease in body fat, improved glucose tolerance and insulin sensitivity, and are protected against hepatic steatosis. Moreover, these mice exhibit a significant increase in fat lipolysis, consistent with their dramatic reduction in adiposity. Rescue experiments that block hepcidin up-regulation and restore iron levels in *Tmprss6*^*−/*−^ mice via anti-hemojuvelin (HJV) therapy, revert the obesity-resistant phenotype of *Tmprss6*^*−/*−^ mice. Overall, this study describes a role for matritpase-2 and hepcidin in obesity and highlights the relevance of iron regulation in the control of adipose tissue function.

## Abstract (web summary)

Iron homeostasis dysfunctions have been associated with several metabolic disorders including obesity, steatosis and diabetes. Here the authors demonstrate that the hepcidin repressor matriptase-2 regulates adiposity and its deficiency protects mice against obesity and promotes lipolysis.

## Introduction

Hypoferremia is commonly associated with chronic low-grade inflammatory conditions (anemia of inflammation), such as obesity^1^. Poor iron status is attributed to enhanced expression of the main iron regulatory hormone hepcidin in obese condition in response to the inflammatory stimuli^2,3^. Hepcidin blocks iron flux into the plasma by its direct binding to ferroportin, the principal iron exporter localized at the cell membrane of duodenal enterocytes, which absorb iron from the diet, and macrophages, which recycle iron from senescent erythrocytes. Hepcidin binding to ferroportin triggers its internalization and subsequent lysosomal degradation, thereby blocking iron export to the circulatory system^4^. In addition to inflammatory cytokines, high-iron levels also cause hepcidin up-regulation^5^. Importantly, iron overload has been associated with a variety of diseases and disorders frequently associated with obesity, such as hepatic steatosis, diabetes and insulin resistance^6^. Although hepcidin is mainly produced by the liver^7–9^, it has recently been described that other cell types such as adipocytes can produce hepcidin^3^. Moreover, it has been shown that adipocytes also express ferroportin and that in vitro hepcidin treatment leads to its down-regulation in this cell type. Interestingly, the consequences of iron deregulation in adipose tissue have recently been linked to alterations in adipokine secretion and insulin resistance^10–12^.

Previously, we demonstrated that matriptase-2 (encoded by *Tmprss6*), a type II transmembrane serine protease, is a repressor of hepcidin expression^13^. Mice deficient in matriptase-2, or lacking the proteolytic domain of this enzyme (*mask* mice), develop hypochromic microcytic anemia as a result of the abnormally elevated levels of hepcidin expression in the liver and the decreased ferroportin expression in duodenal cells^13–18^. In humans, mutations in *TMPRSS6* cause iron-refractory iron deficiency anemia (IRIDA), characterized by no hematological improvement following treatment with oral iron^19–22^. The primary aim of this study was to investigate whether matriptase-2, a negative regulator of hepcidin, may influence obesity development and its associated pathological conditions. We find that matriptase-2 deficiency protects from high-fat diet-induced obesity by increasing fat lipolysis, a phenotype which is dependent on the inadequate hepcidin up-regulation and iron imbalance characteristic of *Tmprss6*^*−/*−^ mice.

## Results

### Matriptase-2 loss protects against HFD-induced obesit**y**

**Fig. 1:**
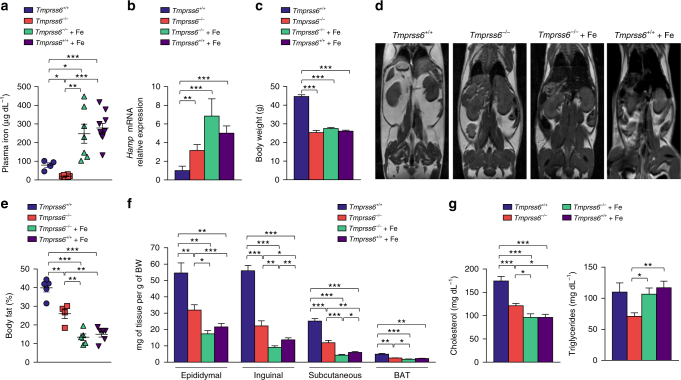
*Tmprss6*-deficient mice are resistant to high-fat diet-induced obesity. **a** Fasting plasma concentration of iron in *Tmprss6*^*+/+*^ (*n* = 4), *Tmprss6*^*−/*−^ (*n* = 8), and both iron-treated *Tmprss6*^*−/*−^ and *Tmprss6*^*+/+*^ (*n* = 7 and *n* = 10, respectively) mice fed a HFD for 20 weeks. **b** Relative gene expression of hepcidin (*Hamp*) in liver samples from HFD-fed *Tmprss6*^*+/+*^ (*n* = 11), *Tmprss6*^*−/*−^ (*n* = 10), and both iron-treated *Tmprss6*^*−/*−^ and *Tmprss6*^*+/+*^ mice (*n* = 10 and *n* = 8, respectively). **c** Body weight of *Tmprss6*^*+/+*^ (*n* = 7), *Tmprss6*^*−/*−^ (*n* = 10), and both iron-treated *Tmprss6*^*−/*−^ and *Tmprss6*^*+/+*^ mice (*n* = 13 and *n* = 10 respectively) fed a HFD for 20 weeks. **d** Representative MRI images across the body of *Tmprss6*^*+/+*^ (*n* = 5), *Tmprss6*^*−/*−^ (*n* = 4), and both iron-treated *Tmprss6*^*−/*−^ and *Tmprss6*^*+/+*^ mice (*n* = 5 and *n* = 6 respectively) fed a HFD. White areas denote fat. **e** Body fat percentage was quantified by image analysis of MRI data on the same mice. **f** White adipose (epididymal, subcutaneous and inguinal) and brown adipose tissue (BAT) masses were determined relative to body weight from HFD-fed *Tmprss6*^*+/+*^ (*n* = 7), *Tmprss6*^*−/*−^ (*n* = 10), and both iron-treated *Tmprss6*^*−/*−^ and *Tmprss6*^*+/+*^ mice (*n* = 12-13 and *n* = 10 respectively). **g** Fasting plasma levels of cholesterol and triglycerides from HFD-fed *Tmprss6*^*+/+*^ (*n* = 7–9), *Tmprss6*^*−/*−^ (*n* = 8–9), and both iron-treated *Tmprss6*^*−/*−^ and *Tmprss6*^*+/+*^ mice (*n* = 9 and *n* = 9–10 respectively). Data shown are mean ± SEM. *P *<* 0.05, **P *<* 0.01, ***P < 0.001, two-tailed Student’s *t* test and Mann–Whitney test

Hepcidin levels have been found to be increased in obese patients^3^. To determine whether the loss of a negative regulator of hepcidin plays a significant role in the development of an obese phenotype, we fed wild-type and *Tmprss6*^−/−^ mice a high-fat diet (HFD) for 20 weeks after weaning. In addition, to evaluate the specific contribution of the hypoferremic phenotype of *Tmprss6*^−/−^ mice in the outcome of the experiment, we treated *Tmprss6*^−/−^ mice with intraperitoneal injections of iron-dextran to revert their plasma iron deficiency. As shown in Supplementary Fig. 1, this treatment resulted in a marked rescue of most of the hematologic parameters characteristic of IRIDA, which are found in *Tmprss6*^*−/−*^ mice. However, iron-treated *Tmprss6*^*−/*−^ mice showed systemic and tissue iron levels above those found in wild-type mice (Fig. 1a and Supplementary Fig. 2 and 11). Therefore, as a control for iron overload in obesity and metabolic analysis, we also treated wild-type mice with intraperitoneal injections of iron-dextran in order to obtain similar iron levels to those observed in iron-treated *Tmprss6*^−*/−*^ mice. Consistent with hepcidin regulation by iron^8,23^, the higher circulating iron levels found in both iron-treated *Tmprss6*^−*/−*^ and *Tmprss6*^*+/+*^ mice further increased hepcidin expression over the abnormally high levels found in the absence of matriptase-2 (Fig. 1b). Interestingly, we observed that all experimental models showing high hepcidin levels (*Tmprss6*^*−/*−^ and iron-treated *Tmprss6*^*−/*−^ mice, as well as iron-treated *Tmprss6*^*+/+*^ mice) showed significantly less body weight than wild-type controls after 20 weeks on a high-fat diet (Fig. 1c). Body weight of iron-treated *Tmprss6*^*−/*−^ and *Tmprss6*^*−/*−^ mice was decreased by 35% when normalized to their length, since the size of *Tmprss6*^*−/*−^ mice is slightly smaller^13^ and it is partially recovered after iron administration (Supplementary Fig. 3a, b). Thus, iron-treated *Tmprss6*^−/−^ and *Tmprss6*^*−/−*^ mice gained 50% less weight than wild-type mice when challenged with a HFD (Supplementary Fig. 3c). The reduction in weight gain was more pronounced in iron-treated *Tmprss6*^*+/+*^ because of their higher weight at the onset of the experiment compared to *Tmprss6*^*−/*−^ and iron-treated *Tmprss6*^*−/*−^ mice (Supplementary Fig. 3c). Moreover, analysis of body composition by MRI showed a significant decrease in the percentage of body fat in *Tmprss6*^*−/*−^ mice, which was further exacerbated by iron administration (Fig. 1d, e). Further, analysis of fat deposits demonstrated that fat pad weights normalized to total body weight was reduced in all compartments analyzed, including brown adipose tissue (BAT), in *Tmprss6*^*−/*−^ mice and in both *Tmprss6*^*−/*−^ and *Tmprs6*^*+/+*^ mice treated with iron compared with wild-type animals (Fig. 1f). In agreement with MRI analyses, the reduction in fat deposits was more pronounced in both iron-treated *Tmprss6*^*−/*−^ and *Tmprss6*^*+/+*^ mice than in *Tmprss6*^*−/*−^ mice. Opposite to fat pads, the weight of other tissues normalized to total body weight was higher in *Tmprss6*^*−/*−^ mice and in both iron-treated *Tmprss6*^*−/*−^ and *Tmprss6*^*+/+*^ mice than in wild-type mice (Supplementary Fig. 3d). Altogether, our results indicate that the reduced weight gain observed in *Tmprss6*^*−/*−^ mice and in both *Tmprss6*^*−/*−^ and *Tmprss6*^*+/+*^ mice treated with iron compared to wild-type mice is attributed to a lower fat content. Moreover, the analysis of lipid levels, frequently altered in obesity, showed a significant reduction in the concentration of plasma cholesterol in *Tmprss6*^*−/*−^ mice and in both *Tmprss6*^*−/*−^ and *Tmprss6*^*+/+*^ mice treated with iron compared with controls (Fig. 1g). However, we found that plasma triglycerides were lower in *Tmprss6*^*−/*−^ mice, but not in both iron-treated *Tmprss6*^*−/*−^ and *Tmprss6*^*+/+*^ mice, compared to wild-type mice (Fig. 1g).

### Reduced adipocyte hypertrophy in the absence of matriptase-2

**Fig. 2:**
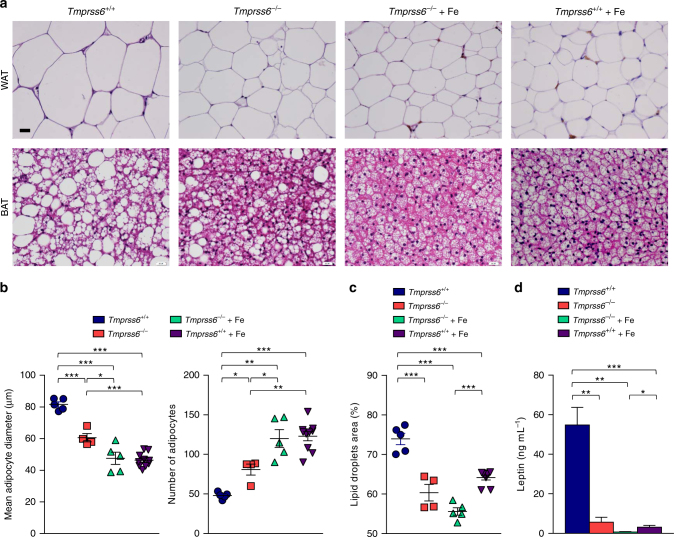
Matriptase-2 deficiency prevents diet-induced adipocyte hypertrophy. **a** Representative images of epididymal fat pad and BAT sections stained with H&E from *Tmprss6*^*+/+*^ (*n* = 5), *Tmprss6*^*−/*−^ (*n* = 4), and both iron-treated *Tmprss6*^*−/*−^ and *Tmprss6*^*+/+*^ mice (*n* = 5 and *n* = 9–10 respectively) fed a HFD for 20 weeks. **b** Size and number of epididymal adipocytes in *Tmprss6*^*+/+*^ (*n* = 5), *Tmprss6*^*−/*−^ (*n* = 4), and both iron-treated *Tmprss6*^*−/*−^ and *Tmprss6*^*+/+*^ mice (*n* = 5 and *n* = 10, respectively). **c** Percentage of total area occupied by all lipid droplets in BAT sections stained with H&E in *Tmprss6*^*+/+*^ (*n* = 5), *Tmprss6*^*−/*−^ (*n* = 4), and both iron-treated *Tmprss6*^*−/*−^ and *Tmprss6*^*+/+*^ mice (*n* = 5 and *n* = 9, respectively). **d** Fasting plasma concentration of leptin in *Tmprss6*^*+/+*^ (*n* = 6), *Tmprss6*^*−/*−^ (*n* = 6), and both iron-treated *Tmprss6*^*−/*−^ and *Tmprss6*^*+/+*^ mice (*n* = 6 and *n* = 9, respectively) fed a HFD. Scale bar=20 µm. Data shown are mean ± SEM. *P *<* 0.05, **P *<* 0.01, ***P < 0.001, two-tailed Student’s *t* test and Mann–Whitney test

To better understand the observed reduction in fat deposits in *Tmprss6*^*−/*−^ mice upon high-fat feeding, we performed histological analyses of white (WAT) and brown adipose tissues. WAT analysis revealed a significant decrease in adipocyte cell size in *Tmprss6*^*−/*−^ mice and in both iron-treated *Tmprss6*^*−/*−^ and *Tmprss6*^*+/+*^ mice compared with wild-type animals (Fig. 2a, b). Accordingly, this reduction in adipocyte hypertrophy was accompanied by a concomitant increase in the number of adipocytes per area (Fig. 2a, b). Moreover, histological examination of BAT also showed a significant reduction in fat content in the absence of matriptase-2. Thus, iron-treated *Tmprss6*^*−/*−^ and *Tmprss6*^*−/*−^mice showed smaller and multilocular lipid droplets compared to wild-type controls (Fig. 2a, c), indicating that the observed resistance to the obesity-induced phenotype in *Tmprss6*^*−/*−^ mice was not only related to a decrease in WAT hypertrophy, but also to a reduction in the lipid content of BAT. This phenotype was also observed in iron-treated *Tmprss6*^*+/+*^ mice (Fig. 2a–c). Remarkably, analysis of the expression levels of genes involved in adipocyte differentiation, such as *Cebpa*, *Srebf1*, and *Pparg*, showed no major differences between wild-type and *Tmprss6*^*−/*−^ mice, suggesting that the observed changes in adipocyte size were not due to alterations in the main adipocyte differentiation program (Supplementary Fig. 4a, b). Of note, the expression of some of these genes was down-regulated in those experimental groups where the reduction in body fat was more exacerbated, as in both *Tmprss6*^*−/*−^ and *Tmprss6*^*+/+*^ mice treated with iron.

To further explore those metabolic parameters that could be influenced by the loss of fat mass, we analyzed the levels of leptin, a key adipokine produced by the adipose tissue. These analyses revealed a striking reduction in plasma leptin concentration in the absence of matriptase-2 and in the iron-treated experimental groups, all of them showing a lean phenotype, compared to wild-type mice (Fig. 2d). Thus, consistent with low leptin levels, *Tmprss6*^*−/*−^ mice and both iron-treated *Tmprss6*^*−/*−^ and *Tmprss6*^*+/+*^ mice showed increased food intake compared with wild-type controls (Supplementary Fig. 3e). Therefore, these results demonstrated that the reduced weight gain observed in the absence of matriptase-2 was not due to a lower appetite.

### Decreased hepatic steatosis in *Tmprss6*^−/−^ mice fed a HFD

**Fig. 3:**
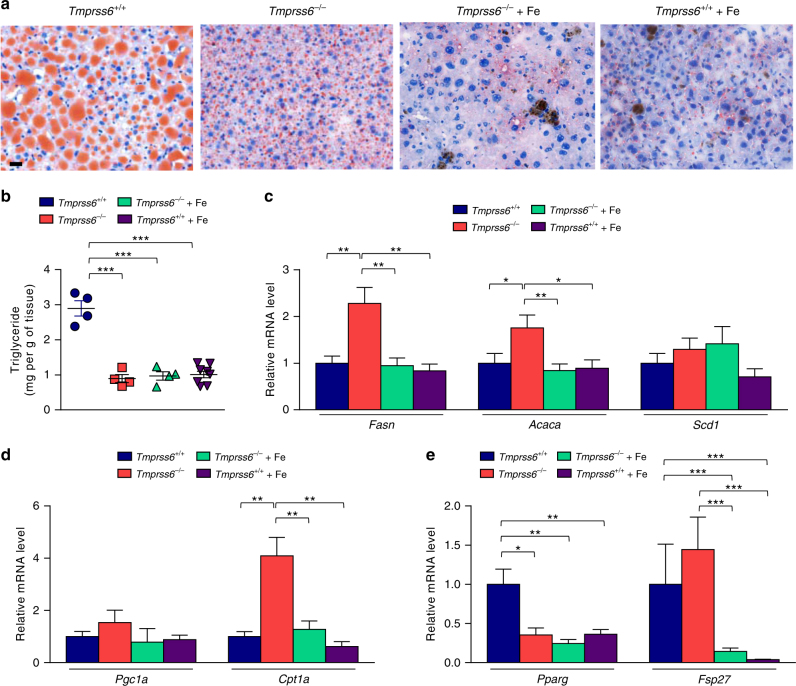
Matriptase-2 loss impairs hepatic steatosis upon HFD feeding. **a** Representative Oil Red O staining of liver sections from *Tmprss6*^*+/+*^ (*n* = 4), *Tmprss6*^*−/*−^ (*n* = 4), and both iron-treated *Tmprss6*^*−/*−^and *Tmprss6*^*+/+*^ mice (*n* = 4 and *n* = 8, respectively) fed a HFD. **b** Triglyceride content of liver samples from HFD-fed *Tmprss6*^*+/+*^ (*n* = 4), *Tmprss6*^*−/*−^(*n* = 4), and both iron-treated *Tmprss6*^*−/*−^ and *Tmprss6*^*+/+*^ mice (*n* = 4 and *n* = 8, respectively). Relative expression levels of genes related to lipogenesis (**c**), β-oxidation (**d**), and lipid storage (**e**) in liver samples from HFD-fed *Tmprss6*^*+/+*^ (*n* = 11), *Tmprss6*^*−/*−^ (*n* = 8–10), and both iron-treated *Tmprss6*^*−/*−^ and *Tmprss6*^*+/+*^ mice (*n* = 9-10 and *n* = 7–8 respectively). Scale bar=20 µm. Data shown are mean ± SEM. *P *<* 0.05, **P *<* 0.01, ***P < 0.001, two-tailed Student’s *t* test and Mann–Whitney test

Next, we analyzed whether matriptase-2 deficiency also prevented the development of hepatic steatosis, a common disease frequently associated with obesity. Thus, Oil Red O staining of liver sections of HFD-fed mice showed a marked macrovesicular pattern of steatosis in wild-type mice (Fig. 3a). However, this macrovesicular pattern was not detected in *Tmprss6*^*−/*−^nor in both iron-treated *Tmprss6*^*−/*−^ and *Tmprss6*^*+/+*^ mice, indicating a clear reduction in liver lipid accumulation in the absence of matriptase-2 (Fig. 3a). Consistent with these findings, quantification of liver triglyceride content showed a significant reduction in triglyceride levels in *Tmprss6*^*−/*−^ mice and in both *Tmprss6*^*−/*−^ and *Tmprss6*^*+/+*^ mice treated with iron compared with wild-type animals (Fig. 3b). To examine whether the observed reduction in lipid content was due to alterations in the hepatic lipid metabolism, we analyzed the expression levels of key genes involved in lipid synthesis and fatty acid oxidation. Surprisingly, we found a significant up-regulation in the expression levels of genes involved in de novo lipid synthesis, such as *Fasn* and *Acaca*, in *Tmprss6*^*−/*−^mice compared to wild-type mice (Fig. 3c). Furthermore, we found that the expression of one of the main regulators of fatty acid oxidation, such it is *Cpt1a*, was also increased in *Tmprss6*^*−/*−^mice (Fig. 3d). Interestingly, this up-regulation was not observed in iron-treated *Tmprss6*^*−/*−^animals, suggesting that the increased expression of diverse metabolic enzymes with opposite functions in the absence of matriptase-2 is mainly caused by their hypoferremic phenotype, since iron treatment completely reverted the observed changes to wild-type levels. Nevertheless, in agreement with the observed histological and biochemical data, we found that the expression level of *Pparg*, a key driver of hepatic triglyceride storage^24^, was markedly down-regulated in *Tmprss6*^*−/*−^ mice and in both iron-treated *Tmprss6*^*−/*−^ and *Tmprss6*^*+/+*^ mice compared to wild-type animals (Fig. 3e). This reduction was accompanied by a decrease in the expression level of the *Pparg* downstream target *Fsp27/CIDEC*, encoding a lipid-droplet associated protein that promotes lipid accumulation, in both iron-treated *Tmprss6*^*−/*−^and *Tmprss6*^*+/+*^ mice compared to wild-type animals (Fig. 3e).

### Improved glucose homeostasis in *Tmprss6*^−*/−*^ mice fed a HFD

**Fig. 4:**
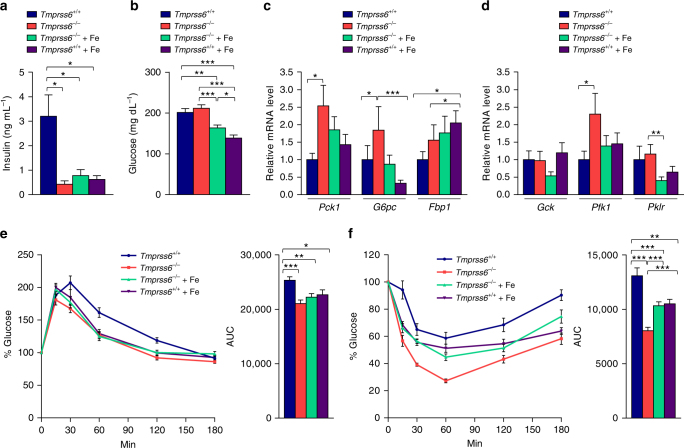
Improved glucose metabolism in HFD-fed *Tmprss6*-deficient mice. **a** Fasting plasma concentration of insulin in HFD-fed *Tmprss6*^*+/+*^ (*n* = 10), *Tmprss6*^*−/*−^(*n* = 6), and both iron-treated *Tmprss6*^−*/*−^ and *Tmprss6*^*+/+*^ mice (*n* = 8 and *n* = 6 respectively). **b** Fasting blood glucose concentration in HFD-fed *Tmprss6*^*+/+*^ (*n* = 11), *Tmprss6*^*−/*−^ (*n* = 13), and both iron-treated *Tmprss6*^*−/*−^and *Tmprss6*^*+/+*^ mice (*n* = 13 and *n* = 10 respectively). Relative expression levels of genes related to gluconeogenesis (**c**) and glycolysis (**d**) in liver samples from HFD-fed *Tmprss6*^*+/+*^ (*n* = 11), *Tmprss6*^−*/*−^ (*n* = 8–9), and both iron-treated *Tmprss6*^*−/*−^and *Tmprss6*^*+/+*^ mice (*n* = 10-11 and *n* = 8 respectively). **e** Glucose tolerance test after overnight fasting in HFD-fed *Tmprss6*^*+/+*^ (*n* = 11), *Tmprss6*^*−/*−^ (*n* = 12), and both iron-treated *Tmprss6*^*−/*−^and *Tmprss6*^*+/+*^ mice (*n* = 9 and *n* = 9 respectively). **f** Insulin tolerance test after overnight fasting in HFD-fed *Tmprss6*^*+/+*^ (*n* = 11), *Tmprss6*^*−/*−^ (*n* = 12), and both iron-treated *Tmprss6*^*−/*−^and *Tmprss6*^*+/+*^ mice (*n* = 13 and *n* = 11 respectively). AUC, area under the curve. Data shown are mean ± SEM. *P *<* 0.05, **P *<* 0.01, ***P < 0.001, two-tailed Student’s *t* test and Mann–Whitney test

The striking resistance to the development of an obese phenotype observed in the absence of matriptase-2, prompted us to evaluate whether matriptase-2 deficiency also affected glucose homeostasis. Thus, consistent with the reduced fat content, we found lower plasma insulin levels in *Tmprss6*^−*/−*^ mice and in both iron-treated *Tmprss6*^*−/*−^and *Tmprss6*^*+/+*^ mice compared with wild-type mice, suggesting the possibility of improved insulin sensitivity in the absence of matriptase-2 (Fig. 4a). Accordingly, the concentration of fasting blood glucose was significantly reduced in both iron-treated *Tmprss6*^*−/*−^and *Tmprss6*^*+/+*^ mice, in which the resistance to diet-induced obesity was more exacerbated, but not in *Tmprss6*^*−/*−^mice compared with wild-type mice (Fig. 4b). Interestingly, analysis of the expression levels of key hepatic gluconeogenic and glycolytic genes, such as *Pck1* and *Pfk1*, revealed a significant up-regulation of these genes in *Tmprss6*^*−/*−^mice compared to those found in wild-type mice (Fig. 4c, d). However, similarly to what we observed in the analysis of genes involved in the regulation of lipid metabolism, this up-regulation of enzymes belonging to opposite metabolic routes was ameliorated or completely reverted in iron-treated *Tmprss6*^*−/*−^ mice (Fig. 4c, d). Altogether, these results suggest that the observed deregulation in the expression levels of both gluconeogenic and glycolytic genes in *Tmprss6*^*−/*−^mice is a consequence of their iron deficiency, which may explain why glucose levels are not decreased in these mice compared with wild-type animals despite their leaner phenotype. Nevertheless, to further explore glucose homeostasis in the absence of matriptase-2, we performed glucose tolerance tests by intraperitoneal administration of glucose to the mice. These experiments revealed a significant improved glucose clearance in *Tmprss6*^*−/*−^and iron-treated *Tmprss6*^*−/*−^mice compared with wild-type animals (Fig. 4e). Similarly, blood glucose levels decreased more rapidly in knock-out mice upon insulin injection, indicating increased insulin sensitivity in these mice, which is consistent with their lower insulin levels (Fig. 4f). Remarkably, iron treatment partially increased insulin resistance of *Tmprss6*^*−/*−^mice, although this resistance did not reach wild-type levels. Similar results were also observed in iron-treated *Tmprss6*^*+/+*^ mice. These findings are in agreement with a deleterious role of iron on adipose tissue function^10^. Altogether, these results demonstrate that matriptase-2-deficiency protects against the development of glucose intolerance and insulin resistance associated with diet-induced obesity, probably as a consequence of their decreased fat mass.

### Increased fat lipolysis in *Tmprss6*^*−/*−^ mice fed a HFD

**Fig. 5:**
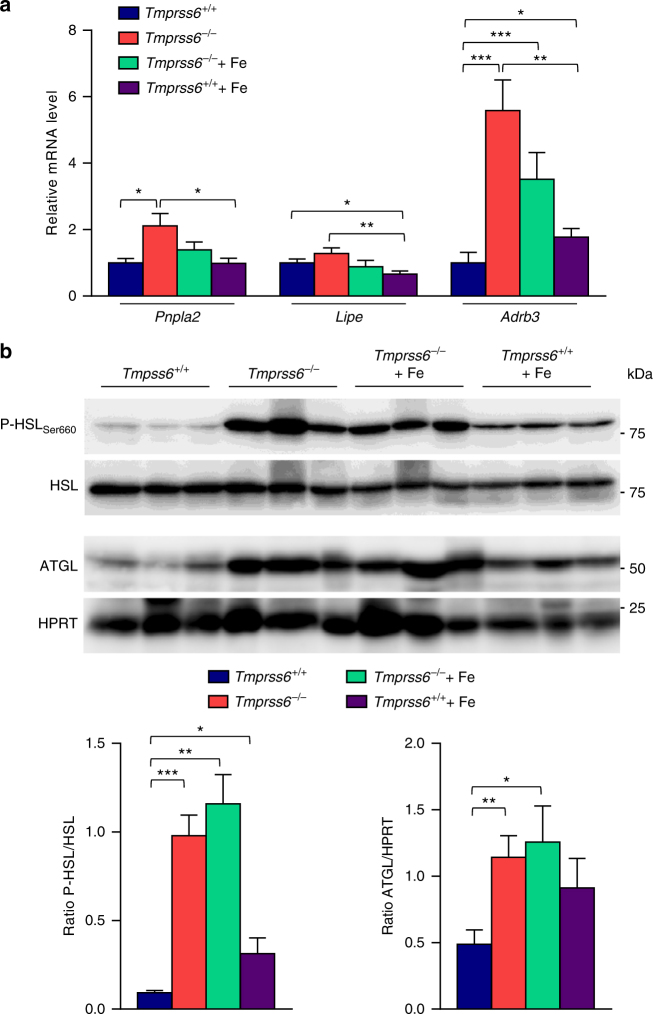
Increased WAT lipolysis in mice lacking matriptase-2 upon HFD feeding. **a** Relative expression levels of genes related to lipolysis in WAT samples from HFD-fed *Tmprss6*^*+/+*^ (*n* = 11), *Tmprss6*^*−/*−^(*n* = 7), and both iron-treated *Tmprss6*^*−/*−^and *Tmprss6*^*+/+*^ mice (*n* = 11 and *n* = 8, respectively). **b** Western-blot analysis of phospho-HSL(Ser660), total HSL and ATGL protein expression in WAT samples from HFD-fed *Tmprss6*^*+/+*^ (*n* = 6), *Tmprss6*^*−/*−^(*n* = 6), and both iron-treated *Tmprss6*^*−/*−^and *Tmprss6*^*+/+*^ mice (*n* = 6 and *n* = 7 respectively). (top) A representative result showing increased phospho-HSL and ATGL protein levels in *Tmprss6*^*−/−*^ and both iron-treated *Tmprss6*^*−/*−^and *Tmprss6*^*+/+*^ mice compared to wild-type mice. (bottom) Quantification of phospho-HSL protein levels relative to total HSL levels and ATGL protein levels relative to loading control HPRT (hypoxanthine-guanine phosphoribosyltransferase). Note that phospho-HSL and the corresponding loading control, the total HSL protein, were detected on the same samples run in parallel in two identical blots. Data shown are mean ± SEM. *P *<* 0.05, **P *<* 0.01, ***P < 0.001, two-tailed Student’s *t* test and Mann–Whitney test

To determine the possible causes of the reduced adiposity observed in the absence of matriptase-2, we next analyzed mice energy expenditure by using indirect calorimetry. These experiments showed higher rates of oxygen consumption and CO_2_ production adjusted for lean mass in *Tmprss6*^*−/*−^mice compared with wild-type mice during both light and dark phases (Supplementary Fig. 5a, b). Accordingly, energy expenditure was significantly increased in these mice (Supplementary Fig. 5c). Remarkably, iron treatment completely reverted this phenotype. Thus, iron-treated *Tmprss6*^*−/*−^mice showed similar energy expenditure than wild-type mice (Supplementary Fig. 5c), suggesting that the observed alterations in the absence of matriptase-2 could be a consequence of the hypoferremic phenotype. These changes occurred in the absence of a significant increase in the locomotor activity or the expression levels of thermogenic genes in BAT samples of *Tmprss6*^−/−^ mice compared to those found in wild-type mice (Supplementary Fig. 5d, 6). Taken together, these data suggest that energy balance might not be the main mechanism responsible for the observed resistance to diet-induced obesity in the absence of matriptase-2, since iron-treated *Tmprss6*^*−/*−^mice behave similar to wild-type mice in this regard but show a marked lean phenotype. Therefore, to get further insights into the mechanisms that could explain the reduced fat content of *Tmprss6*^*−/*−^and iron-treated *Tmprss6*^*−/*−^mice, we analyzed the expression levels of the main genes responsible for the hydrolysis of stored lipids in adipocytes in WAT samples. Notably, we found a significant increase in *Pnpla2* (adipose triglyceride lipase, ATGL) gene expression in *Tmprss6*^*−/*−^mice compared to those in wild-type mice (Fig. 5a), which was further confirmed at the protein level (Fig. 5b and Supplementary Fig. 11). ATGL protein concentration was also significantly elevated in iron-treated *Tmprss6*^*−/*−^mice (Fig. 5b and Supplementary Fig. 11). No differences were detected in the expression level of the lipolytic gene *Lipe* (hormone-sensitive lipase, HSL), but we observed a significant increase in the expression level of *Adrb3* (β3-adrenergic receptor) in *Tmprss6*^*−/*−^and iron-treated *Tmprss6*^*−/*−^mice compared to wild-type mice (Fig. 5a), being β-adrenergic signaling one of the main pathways responsible for the control of HSL activity and fat mobilization in the adipose tissue^25^. Thus, β-adrenergic stimulation leads to the phosphorylation of HSL, which increases the hydrolytic activity of the enzyme against triacylglycerol substrate^26^. Considering that HSL is primarily regulated by post-translational mechanisms rather than at the mRNA level, we were prompted to analyze the phosphorylation status of HSL in WAT samples. Western-blot analysis revealed a striking increase in phospho-HSL levels versus total HSL protein in *Tmprss6*^*−/*−^and iron-treated *Tmprss6*^*−/*−^mice compared with wild-type mice (Fig. 5b and Supplementary Fig. 11). Interestingly, HSL phosphorylation was also significantly elevated in iron-treated *Tmprss6*^*+/+*^ mice (Fig. 5b and Supplementary Fig. 11).

Taken together, these results suggest that the reduced adiposity observed in matriptase-2 deficient mice, with and without iron supplementation, is due to an increased lipolysis triggered by the two main lipolytic enzymes, HSL and ATGL. In addition, we found that HSL phosphorylation and ATGL concentration were increased in iron-treated *Tmprss6*^*+/+*^ mice, suggesting a relevant role for the lipolytic pathway in these mice that also show a significant reduction in their fat mass compared to wild-type mice.

### Hepcidin down-regulation induces obesity in *Tmprss6*^*−/*−^ mice

Considering that adipocyte iron deregulation has recently been associated to alterations in adipokine secretion and insulin resistance^10–12^, we next asked if variations in adipocyte iron content may also influence the lipolytic program of the adipose tissue of matriptase-2 deficient mice. To this end, we measured iron levels in adipocytes isolated from WAT samples obtained from all experimental groups. No differences were observed in the iron content of adipocytes from *Tmprss6*^*−/*−^and iron-treated *Tmprss6*^*−/*−^mice compared to wild-type mice. Remarkably, the excess of iron of both *Tmprss6*^*−/*−^and *Tmprss6*^*+/+*^ mice treated with iron was mostly accumulated in the stromal-vascular fraction (SVF) of the adipose tissue, which contains a high percentage of infiltrating macrophages, rather than in the adipocytes themselves (Supplementary Fig. 7).

**Fig. 6:**
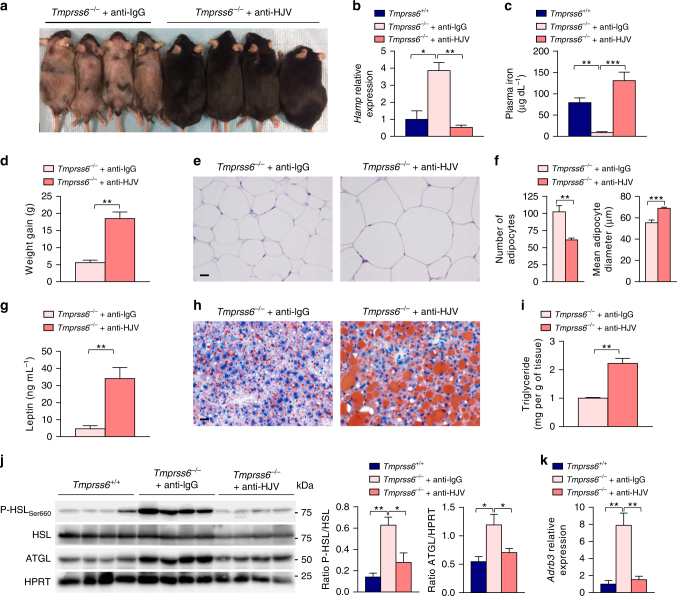
Hepcidin down-regulation in *Tmprss6*-deficient mice reverts the obesity-resistant phenotype. **a** Representative image of HFD-fed *Tmprss6*^*−/*−^ mice treated weekly with anti-HJV neutralizing antibody or control anti-IgG for 18 weeks. The image shows the hair coat recovery and the increased body weight of anti-HJV-treated *Tmprss6*^*−/*−^ mice compared to *Tmprss6*^*−/*−^ mice treated with control anti-IgG. **b** Relative gene expression of hepcidin (*Hamp*) in liver samples from HFD-fed *Tmprss6*^*+/+*^ (*n* = 7), anti-IgG-treated *Tmprss6*^*−/*−^ (*n* = 5), and anti-HJV-treated *Tmprss6*^*−/*−^ (*n* = 8) mice. **c** Fasting plasma concentration of iron in HFD-fed *Tmprss6*^*+/+*^ (*n* = 4), anti-IgG-treated *Tmprss6*^−/−^ (*n* = 5), and anti-HJV-treated *Tmprss6*^*−/*−^ (*n* = 8) mice. **d** Weight gain of anti-IgG-treated *Tmprss6*^*−/*−^ (*n* = 5), and anti-HJV-treated *Tmprss6*^−/−^(*n* = 8) HFD-fed mice after 18 weeks of antibody administration. **e** Representative images of epididymal fat pad sections stained with H&E from HFD-fed anti-IgG-treated *Tmprss6*^*−/*−^ (*n* = 5) and anti-HJV-treated *Tmprss6*^*−/*−^ (*n* = 8) mice. **f** Number and size of epididymal adipocytes in HFD-fed anti-IgG-treated *Tmprss6*^−/−^(*n* = 5), and anti-HJV-treated *Tmprss6*^−/−^(*n* = 8) mice. **g** Fasting plasma concentration of leptin in anti-IgG-treated *Tmprss6*^−/−^(*n* = 5), and anti-HJV-treated *Tmprss6*^*−/*−^ (*n* = 8) mice fed a HFD. **h** Representative Oil Red O staining of liver sections from anti-IgG-treated *Tmprss6*^*−/*−^ (*n* = 5), and anti-HJV-treated *Tmprss6*^−/−^(*n* = 8) mice fed a HFD. **i** Triglyceride content of liver samples from HFD-fed anti-IgG-treated *Tmprss6*^−/−^(*n* = 5), and anti-HJV-treated *Tmprss6*^−/−^(*n* = 8) mice. **j** Western-blot analysis of phospho-HSL(Ser660), total HSL and ATGL protein expression in WAT samples from HFD-fed *Tmprss6*^*+/+*^ (*n* = 5), anti-IgG-treated *Tmprss6*^−/−^(*n* = 5), and anti-HJV-treated *Tmprss6*^−/−^(*n* = 8) mice. (left) A representative result showing decreased phospho-HSL and ATGL protein levels in anti-HJV-treated *Tmprss6*^*−/*−^ mice compared to anti-IgG-treated *Tmprss6*^−/−^mice. (right) Quantification of phospho-HSL protein levels relative to total HSL levels and ATGL protein levels relative to loading control HPRT. Note that phospho-HSL and the corresponding loading control, the total HSL protein, were detected on the same samples run in parallel in two identical blots. **k** Relative gene expression of *Adrb3* in WAT samples from HFD-fed *Tmprss6*^*+/+*^ (*n* = 8), anti-IgG-treated *Tmprss6*^−/−^(*n* = 5), and anti-HJV-treated *Tmprss6*^−/−^(*n* = 8) mice. Scale bar 20 µm. Data shown are mean ± SEM. *P *<* 0.05, **P *<* 0.01, ***P < 0.001, two-tailed Student’s *t* test and Mann–Whitney test

On the basis of the high hepcidin levels found in the three experimental groups with a leaner phenotype, *Tmprss6*^*−/*−^and both iron-treated *Tmprss6*^*−/*−^and *Tmprss6*^*+/+*^ mice, we further investigated whether hepcidin up-regulation might be responsible for the induction of lipolysis and the HFD-obesity resistance phenotype observed in matriptase-2 deficient mice. To test this hypothesis, we aimed at blocking hepcidin up-regulation in *Tmprss6*^*−/*−^mice by using a neutralizing antibody against hemojuvelin (HJV), a molecule that acts as a co-receptor of the BMP (bone morphogenetic protein) signaling pathway that promotes hepcidin transcription^27^. We treated weekly *Tmprss6*^*−/*−^mice with an anti-HJV neutralizing antibody or control anti-IgG for 18 weeks. Thus, mice were treated for the first time at the age of 6 weeks, two weeks after weaning and the onset of HFD administration, and the treatment lasted until the time of sacrifice. As shown in Supplementary Fig. 8, the monitoring of plasma hematologic parameters over time showed a rapid rescue of Hgb levels in anti-HJV-treated *Tmprss6*^*−/*−^mice compared to those injected with control anti-IgG after two weeks of treatment. This recovery was also observed in the rest of hematologic parameters analyzed, which peaked 4 weeks after the onset of the antibody therapy and it was sustained during the whole treatment period, reaching wild-type levels in anti-HJV-treated *Tmprss6*^*−/*−^mice (Table 1). Consistent with these data, anti-HJV therapy also restored the hair coat of *Tmprss6*^*−/*−^mice as a result of the recovery of plasma iron levels^13^ (Fig. 6a, c). The analysis of liver samples from this experimental setting allowed us to confirm the significant reduction of hepcidin expression in anti-HJV-treated *Tmprss6*^*−/*−^ mice compared to anti-IgG-treated *Tmprss6*^*−/−*^ mice, thereby demonstrating the efficacy of the anti-HJV therapy (Fig. 6b). Consistent with the restoration of systemic iron levels upon hepcidin down-regulation, the concentration of plasma ferritin significantly increased in anti-HJV-treated *Tmprss6*^*−/*−^mice compared to anti-IgG-treated *Tmprss6*^*−/*−^ mice, reaching wild-type levels (Supplementary Fig. 9a). In agreement, we observed a significant increase in the liver iron content of anti-HJV-treated *Tmprss6*^*−/*−^ mice compared to the anti-IgG control group, which was accompanied by a down-regulation of the transferrin receptor 1 (*Tfrc*, TfR1) mRNA levels and protein expression in this tissue (Supplementary Fig. 9b–d and 11). More important, hepcidin down-regulation in anti-HJV-treated *Tmprss6*^*−/*−^mice completely abolished the obesity-resistant phenotype observed in matriptase-2 deficient mice on HFD diet. Thus, anti-HJV-treated *Tmprss6*^*−/*−^mice gain significantly more weight than anti-IgG-treated *Tmprss6*^*−/*−^mice (Fig. 6d). Further, analysis of fat deposits in these mice demonstrated that the weight gain was due to a significant increase in the adiposity of anti-HJV-treated *Tmprss6*^*−/*−^*versus* anti-IgG-treated mice (Supplementary Fig. 10). This increase in the adiposity was accompanied by a marked adipocyte hypertrophy of WAT samples and a significant increase in leptin production in anti-HJV-treated *Tmprss6*^*−/*−^mice compared to anti-IgG treated *Tmprss6*^*−/*−^mice (Fig. 6e–g). Hepdicin down-regulation also resulted in a marked liver steatosis in anti-HJV-treated *Tmprss6*^*−/*−^mice (Fig. 6h, i). Furthermore, we found that hepdicin down-regulation in matriptase-2 deficient mice through anti-HJV therapy completely reverted the lipolytic program of the adipose tissue to wild-type levels (Fig. 6j, k and Supplementary Fig. 11)

**Table 1:** Hematologic parameters of HFD-fed *Tmprss6*^*+/+*^ and *Tmprss6*^*−/−*^ mice treated with anti-IgG or anti-HJV antibodies

	Hgb (g dL^−1^)	Hct (%)	MCV (fL)	MCH (pg)	MCHC (g dL^−1^)	RDW (%)
*Tmprss6* ^*+/+*^	11.9 ± 0.4	35.1 ± 0.8	39.5 ± 0.4	13.4 ± 0.1	33.9 ± 0.4	18.8 ± 0.2^‡‡‡^
*Tmprss6*^*−/*−^ + anti-IgG	7.8 ± 0.3^***^	24.5 ± 0.8^***^	24.2 ± 0.5^***^	7.7 ± 0.2^***^	31.7 ± 0.7^*^	29.8 ± 0.7^***^
*Tmprss6*^*−/*−^ + anti-HJV	12.2 ± 0.5^†††^	36.5 ± 1.5^†††^	40.5 ± 0.5^†††^	13.5 ± 0.3^†††^	33.5 ± 0.6	21.2 ± 0.6^††^

Complete blood counts were measured from whole blood of HFD-fed *Tmprss6*^*+/+*^ (*n* = 8), *Tmprss6*^−/−^+anti-IgG (*n* = 5) and *Tmprss6*^−/−^+anti-HJV (*n* = 8) male mice after 18 weeks of antibody therapy upon overnight fasting

Iron homeostasis dysfunctions have been associated with several metabolic disorders including obesity, steatosis, and diabetes. Here the authors demonstrate that the hepcidin repressor matriptase-2 regulates adiposity and its deficiency protects mice against obesity and promotes lipolysis

Data are presented as mean±SEM

*Hgb* hemoglobin, *Hct* hematocrit, *MCV* mean corpuscular volume, *MCH* mean corpuscular hemoglobin, *MCHC* mean corpuscular hemoglobin concentration, *RDW* red cell distribution width

^*^P < 0.05; ^***^P < 0.001, two-tailed Student’s *t* test and Mann–Whitney test *Tmprss6*^−*/*−^+anti-IgG *versus Tmprss6*^*+/+*^ mice

^††^*P* < 0.01; ^†††^*P* < 0.001two-tailed Student’s *t* test and Mann–Whitney test *Tmprss6*^*−/*−^+anti-HJV *versus Tmprss6*^*−/*−^+anti-IgG mice

^‡‡‡^*P* < 0.001, two-tailed Student’s *t* test and Mann–Whitney test *Tmprss6*^*−/−*^+anti-HJV versus* Tmprss6*^*+/+*^ mice

Altogether, these data yield insights into the mechanism responsible for the obesity-resistant phenotype of matripase-2 deficient mice, providing functional evidence of the prominent role of hepcidin in the regulation of lipid metabolism.

## Discussion

The role of iron and its main regulatory hormone hepcidin in obesity is still unclear. On the one hand, epidemiological studies have associated elevated hepcidin levels and iron deficiency with obesity^3^. These studies suggest that increased hepcidin concentrations occur as a result of the chronic inflammatory condition linked to obese individuals, which ultimately leads to defective intestinal iron absorption and anemia^2^. On the other hand, iron overload, which also triggers hepcidin up-regulation, has been associated with fatty-liver disease, diabetes and insulin resistance, three common conditions frequently associated with obesity^28,29^. In this work, we investigated whether the in vivo deletion of a negative regulator of hepcidin, matriptase-2, contributes to the development of obesity and its pathogenic features. Thus, we challenged mice deficient in matriptase-2, whose phenotype is characterized by the anemia caused by the inadequate hepcidin up-regulation, with a protocol of diet-induced obesity. Further, we treated *Tmprss6*^−/−^mice with iron in order to determine the specific contribution of iron deficiency in the development of obesity within the same mouse model. However, this treatment resulted in higher systemic and tissue iron levels in iron-treated *Tmprss6*^−/−^mice compared to wild-type mice. Therefore, as a control for iron overload in the analysis of obesity and other metabolic parameters, we also treated wild-type mice with intraperitoneal injections of iron-dextran in order to obtain similar systemic and tissue iron levels as those observed in iron-treated *Tmprss6*^−/−^mice. A summary of the HFD-induced phenotypes of the different mouse models of iron imbalance and hepcidin up-regulation generated in this work is shown in Supplementary Table 1.

Our results demonstrate that both *Tmprss6*^−/−^and iron-treated *Tmprss6*^−/−^mice show a striking protection against body fat accumulation. Moreover, matriptase-2 deficiency also prevents the development of hepatic steatosis, glucose intolerance and insulin resistance, all pathogenic processes associated with obesity. The relationship among hepcidin, iron overload and fatty-liver disease is still poorly understood. In humans, mutations in the *HFE* gene result in low hepcidin production and iron overload, being the most frequent form of hereditary hemochromatosis (HH)^30^. A strong association has been found between patients with HH and nonalcoholic fatty-liver disease (NAFLD)^31,32^. Also, mice deficient in *Hfe* fed a HFD develop hepatic steatosis^33^. However, recent studies performed with hepcidin knock-out mice, which have a hepatic iron overload phenotype, show opposite results. Thus *Hamp1*^*−/*−^ mice develop attenuated steatosis compared to wild-types but increased liver fibrosis when fed a high fat and sucrose diet^34^. In this context, we found that both *Tmprss6*^−/−^mice, which display a marked hypoferremic phenotype due to inadequate hepcidin upregulation, and iron-treated *Tmprss6*^−/−^mice, which are no longer hypoferremic but still preserve high hepcidin levels, were protected against the development of hepatic steatosis. Furthermore, iron-treated wild-type mice, which also showed a significant hepcidin upregulation as a consequence of the exogenous iron administration, were equally protected against lipid accumulation in the liver. The fact that elevated hepcidin levels was the common mechanism shared by the three experimental groups, argued for a putative role of this peptide in the protection against the development of hepatic steatosis. Consistent with this hypothesis, our data demonstrate that blocking hepcidin up-regulation in *Tmprss6*^−/−^mice via anti-HJV therapy, completely reverted hepatic steatosis resistance in matriptase-2 deficient mice. The efficacy of hepcidin down-regulation through the targeting of the BMP receptor signaling pathway had been previously demonstrated with the use of our mouse model^18,35,36^, which represents a valuable tool to test new therapies for patients suffering from anemia associated with inappropriate high hepcidin levels. However, further studies are needed to determine if hepcidin up-regulation alone is responsible for the observed phenotypes in the liver of iron-treated mice or whether additional effects derived from iron deposition may account for the reduced hepatic steatosis observed under iron overload conditions.

The finding that a striking protection against body fat accumulation was observed in the absence of matriptase-2 also supports the hypothesis of an indirect effect of this enzyme in the regulation of this process, since matriptase-2 is not expressed in the adipose tissue. To date, besides clinical correlations between hepcidin/iron and obesity, very few studies have analyzed in vivo the specific role of these factors on the metabolism of fat cells. Recent findings have shown that both leptin and adiponectin production are affected by the increase in iron content of the adipose tissue. Thus, adipocyte specific ferroportin knock-out mice showed a significant reduction in both leptin and adiponectin plasma levels^11,12^. Conversely, *Hfe*^*−/*−^ mice, with decreased hepcidin levels and increased adipocyte ferroportin expression, showed enhanced leptin production^12^. Although we have not found significant differences in the iron content of the adipocytes isolated from the different experimental groups, since most of the iron deposition was detected in the SVF fraction of the adipose tissue, the finding of reduced leptin production in our mouse models with elevated hepcidin levels are compatible with the above-mentioned reported data. Furthermore, mice fed an iron-enriched diet showed a strong hepcidin up-regulation in the adipose tissue and reduced visceral fat mass and insulin resistance^10^. Interestingly, *Tmprss6*^−/−^mice, which show a mild hepcidin up-regulation, displayed improved insulin sensitivity compared to wild-type mice, potentially accounting for their reduced adiposity. However, in agreement with previous data, both iron-treated *Tmprss6*^−/−^and *Tmprss6*^*+/+*^ mice, which show higher levels of hepcidin compared to untreated *Tmprss6*^−/−^mice in addition to an iron overload phenotype, are more resistant to insulin signaling.

Our results suggest that the reduced adiposity observed in the absence of matriptase-2 is mainly due to the enhanced lipolysis we detected in the adipose tissue of these mice, since no differences were observed in the expression levels of genes involved in adipocyte differentiation or thermogenesis compared with wild-types. Thus, the enhanced energy expenditure observed in the absence of matriptase-2 was completely reverted to wild-type levels upon iron-administration, indicating an important effect of anemia in the control of energy balance. Further, it has been shown that mice treated with an oral iron chelator show higher rates of oxygen consumption and carbon dioxide production, although this treatment was not sufficient to cause a significant effect in body weight^37^. Therefore, altogether, these data point to lipolysis as the main cause responsible for the striking fat mass reduction observed in the absence of matriptase-2, independently of their hypoferremic phenotype. Moreover, the down-regulation of the lipolytic program to wild-type levels, together with the recovery of the fat mass and the increased weight gain observed in anti-HJV-treated *Tmprss6*^−/−^mice, further supports the promoting role of hepcidin and lipolysis in the obesity-resistant phenotype of matriptase-2 deficient mice. We have also considered the possibility that targeting the BMP receptor pathway via anti-HJV therapy may also down-regulate the expression of other BMP targets, such as *Id1*, that are increased in *Tmprss6*^−/−^mice^18^. However, different in vivo models have shown the promoting role of Id proteins in the development of obesity and fat accumulation^38–41^, thus ruling out a significant role of these proteins in the obesity-protection observed in *Tmprss6*^−/−^mice.

In conclusion, our study demonstrates that the loss of matriptase-2 triggers lipolysis and prevents body fat accumulation upon nutrition overload. In addition, we found that the absence of matriptase-2 also protects against obesity-associated pathological conditions such as hepatic steatosis and insulin resistance. Furthermore, we demonstrate that this phenotype is dependent on the inadequate hepcidin up-regulation characteristic of *Tmprss6*^−/−^mice. Thus, anti-HJV-based therapy, which restored hepcidin expression to wild-type levels, completely reverted the obesity-resistant phenotype of matriptase-2 deficient mice. Our findings provide new insights regarding the role of hepcidin and iron regulation in metabolic homeostasis and adipocyte function, and suggest new strategies based on matriptase-2 inhibition for the treatment of obesity.

## Methods

### Animal experiments

*Tmprss6*^−/−^mice have been previously described^13^. *Tmprss6*^−/−^mice were backcrossed 9 generations to C57BL/6N background. Mouse experiments were approved by the Ethics Committee for Animal Experimentation of the Universidad de Oviedo. Iron-treated *Tmprss6*^−/−^mice were injected intraperitoneally with 120 µg per g B.W. of iron-dextran (Sigma) for 12 weeks every other week, starting on postnatal day 15, whereas iron-treated *Tmprss6*^*+/+*^ mice were injected intraperitoneally with 40 µg per g B.W. of iron-dextran for 20 weeks every other week, starting on postnatal day 15. For diet-induced obesity, 4-week-old *Tmprss6*^−/−^, iron-treated *Tmprss6*^−/−^and wild-type male mice were fed a high-fat diet (HFD) containing 60% fat (Harlan D12492) for 20 weeks after weaning. If male mice displayed fight wounds in the course of the experiments they were removed from the experimental cohort according to pre-established exclusion criteria. No statistical method was used to estimate sample size. Body composition was analyzed by magnetic resonance imaging (MRI), using a MR Solutions MRI scanner. For the experiments based on antibody administration, 6-week-old male *Tmprss6*^−/−^mice under a HFD diet were injected intravenously once per week with 20 mg kg^−1^ of anti-hemojuvelin (h5F9-AM8) or anti-IgG antibodies^36^ for 18 weeks. Animals were randomly allocated to experimental groups. Antibodies were dissolved in a buffer containing 30 mM histidine, pH 6.0, 8% w/v sucrose and 0.02% Tween 80. The injection volume administered to the mice was always below 100 µL. To monitor the efficacy of the antibody treatment, 100 µL of blood were extracted directly from the mandibular sinus after anesthetizing the mice with isoflurane at the indicated time points in the text.

### Blood and plasma parameters

For hematological determinations, complete blood counts were analyzed using Abacus junior vet equipment (Diatron labs). For blood glucose determination, blood samples were obtained from the tail vein and measured with Accu-Chek glucometer (Roche Diagnostics). For all the other measurements, blood was extracted directly from the heart after anaesthetizing the mice and collected into heparinized or EDTA-coated tubes. Blood was centrifuged at 1000 × *g* at 4 °C, and the supernatant was stored at −80 °C until analysis. Levels of iron, cholesterol, and triglycerides were determined in IDEXX Laboratorios (Barcelona, Spain). Plasma insulin levels were quantified by using Millipore ELISA Kits, following manufacturer’s instructions. Plasma leptin levels were measured by using R&D ELISA Kits, according to the manufacturer’s protocol. Plasma ferritin levels were determined by using Abcam ELISA Kits, following manufacturer’s instructions. All biochemical parameters above-mentioned were determined in plasma samples obtained from HFD-fed mice upon overnight fasting.

Glucose and insulin tolerance test: Prior to studies, mice were fasted overnight. For glucose tolerance test, mice received an intraperitoneal injection of glucose of 1 g per kg of body weight. For insulin tolerance test, mice received an intraperitoneal injection of 0.75 IU of insulin per kg of body weight. Blood glucose levels were determined with a glucometer as described above. Areas under the curve were calculated using GraphPad Prism 6.0 software.

Determination of liver triglyceride content: Liver triglycerides were determined by using the EnzyChrom Triglyceride Assay Kit from BioAssay Systems (ETGA-200) following manufacturer’s instructions. For sample preparation, 50 mg of tissue were homogenized in 500 µL of 5% Triton X-100. Once homogenized, samples were introduced in a water bath at 80 °C and left inside for 5 min allowing them to reach 100 °C in the water bath. We repeated this procedure twice, allowing the samples to settle at room temperature between cycles. After that, samples were centrifuged for 5 min at 13,000 rpm. The supernatant was recovered and diluted 8-fold in MilliQ water. 10 µL of this dilution was used for the assay. Samples obtained from anti-HJV-treated *Tmprss6*^*−/−*^ and wild-type mice required a further dilution prior the assay.

Adipocyte and SVF isolation: Adipocytes and stromal-vascular fractions (SVF) were isolated from epididymal white adipose tissue samples. 100 mg of this tissue were minced with surgical scissors and incubated at 37 °C for 45 min with constant agitation in Hank’s Balanced Salt Solution (HBSS) containing 2 mg mL^−1^ of collagenase I and II (Sigma) and 2% bovine serum albumin. After digestion, samples were centrifuged for 10 min at 300 × *g* in conical tubes at 4 °C. The upper fat layer containing floating adipocytes was collected into a separated tube and washed with HBSS buffer. The pellet containing the stromal-vascular fraction was resuspended in HBSS buffer and filter through 100 µm nylon mesh. Both fractions were centrifuged once more for 10 min at 400 × *g* at 4 °C. Isolated adipocytes and SVF fractions were snap-frozen and kept at −80 °C.

Iron quantification: Determination of total iron content in liver, spleen and isolated adipocytes and stromal-vascular fractions obtained from the adipose tissue was carried out by using the Iron Assay Kit from Sigma (MAK025). For sample preparation, 50 mg of liver samples, 10 mg of spleen samples or the isolated fractions from the adipose tissue were gently homogenized in 250 µL of Iron Assay Buffer. The homogenates were centrifuged at 13,000 rpm for 10 min at 4 °C to remove insoluble material. 120 µL of the recovered tissue supernatants were incubated with 5 µL of 1 M SDS for 5 min on ice. After that, samples were centrifuged again for 5 min at 13,000 rpm at 4 °C and 100 µL of the recovered supernatant was used for the iron assay following manufacturer’s instructions. Samples obtained from iron-treated mice required a further dilution prior the assay. Iron content was normalized to total protein content in the sample, which was quantified by the BCA assay (Pierce).

Histological analysis: White and brown adipose tissues were fixed in 4% buffered paraformaldehyde solution and embedded in paraffin by standard procedures. Paraffin sections were stained with hematoxylin and eosin (H&E) for adipose tissue evaluation. The number of adipocytes and their mean diameter were determined in 5 µm tissue sections of epididymal fat pads by computer-assisted image analysis. For each sample, 4 different fields were analyzed and 100 adipocytes were measured. For BAT droplets quantification, pictures from BAT sections stained with H&E were analyzed with Image J software. For lipid detection, liver samples were embedded in Tissue-Tek OCT compound (Sakura Finetechnical) and stored at −80 °C. Samples were sectioned at 10 µm thickness and stained with Oil Red O. Histopathological studies were performed by investigators blinded to group identity.

RNA preparation and real-time quantitative PCR: For real-time quantitative PCR, total RNA was isolated from liver and adipose tissues with Trizol and cDNA was synthesized with the ThermoScript RT-PCR system (Invitrogen). PCR was carried out in triplicate for each sample with 100 ng of cDNA using Taqman PCR Master Mix (Applied Biosystems) or Power SYBR® Green PCR Master Mix (Life Technologies). *Hamp* mRNA levels were quantified using Taqman assays (Applied Biosystems). As endogenous controls, gene expression was normalized to *Actb* in liver samples or *Rn18s* in adipose tissue samples. Primer sequences are listed in Supplementary Table 2.

Western-blot analysis: In total 20 µg of white adipose tissue samples or 50 µg of liver samples were separated in SDS-PAGE gels and transferred in CAPS buffer (10 mM 3-[cyclohexylamino]-1-propanesulfonic acid, pH 10.5, and 20% methanol) onto polyvinylidene difluoride (PVDF) membranes (Millipore). Blots were blocked with 5% nonfat dry milk in TBS-T buffer (20 mM Tris–HCl, pH 7.4, 150 mM NaCl, and 0.05% Tween 20) for 1 h at room temperature and incubated overnight at 4 °C with 5% bovine serum albumin in TBS-T buffer with rabbit polyclonal antibodies against phospho-HSL-Ser660 (Cell Signaling, cat. #4126, 1:4000), HSL (Cell Signaling, cat. #4107, 1:4000), and ATGL (Cell Signaling, cat. #2138, 1:4000); and mouse monoclonal antibodies against HPRT (Santa Cruz, cat# sc-376938, 1:1000), TfR1 (Thermo Fisher, cat#13-6800, 1:1000) and β-actin (Sigma, cat#A5441, 1:20000). Finally, blots were incubated for 2 h at room temperature in 1.5% nonfat dry milk with horseradish peroxidase-coupled secondary antibodies, washed and developed with Immobilon Western Chemiluminescent HRP substrate (Millipore). Chemiluminescent images were acquired with a Fujifilm LAS3000 mini apparatus. Phospho-HSL and the corresponding loading control, the total HSL protein, were detected on the same samples run in parallel in two identical blots, after failed attempts using stripping techniques.

### Statistical analysis

Results are reported by plotting independent data points or as graphic bars with mean±SEM. Data were analyzed for normal distribution using Shapiro–Wilk test. Differences between two groups were compared by parametric two-tailed Student’s *t* test or non-parametric Mann–Whitney test. Statistical tests were performed using SPSS, Microsoft Excel and GraphPad Prism 6.0 softwares. A value of *P* < 0.05 was considered significant. Statistically significant differences are shown with asterisks (*P *<* 0.05, **P *<* 0.01, ***P < 0.001).

### Data availability

Data supporting the findings of this study are available within the paper and its supplementary information files.

## Electronic supplementary material

Supplementary Information
