# Supplementary material for: Matriptase-2 deficiency protects from obesity by modulating iron homeostasis
Source: Nat Commun. 2018 Apr 10;9:1350. doi: 10.1038/s41467-018-03853-1 (PMC5893555; doi:10.1038/s41467-018-03853-1)
Supplement: Supplementary file 1 — Supplementary Information [file 41467_2018_3853_MOESM1_ESM.pdf]

Supplementary Information

Matriptase-2 deficiency protects from obesity by modulating iron homeostasis

Alicia R. Folgueras,[1,2*] Sandra Freitas-Rodríguez,[1,2] Andrew J. Ramsay,[1] Cecilia Garabaya,[1] Francisco Rodríguez,[1] Gloria Velasco[1] & Carlos López-Otín[1*]

Supplementary Methods

Determination of food intake, locomotor activity and energy expenditure. All measurements were performed using the Comprehensive Laboratory Animal Monitoring System (Oxymax CLAMS system by Columbus Instruments) and analyzed following manufacturer's instruction. Mice were housed individually and kept on HFD chow on a 12:12-hour light–dark cycle. Mice were monitored for 48 hours and the first 24 hours were discarded in the analysis, considering them as acclimation period. Rates of oxygen consumption (VO_2), CO2 production (VCO_2) and energy expenditure (EE) were normalized to lean mass. Areas under the curve were calculated using GraphPad Prism 6.0 software.

Supplementary Figures

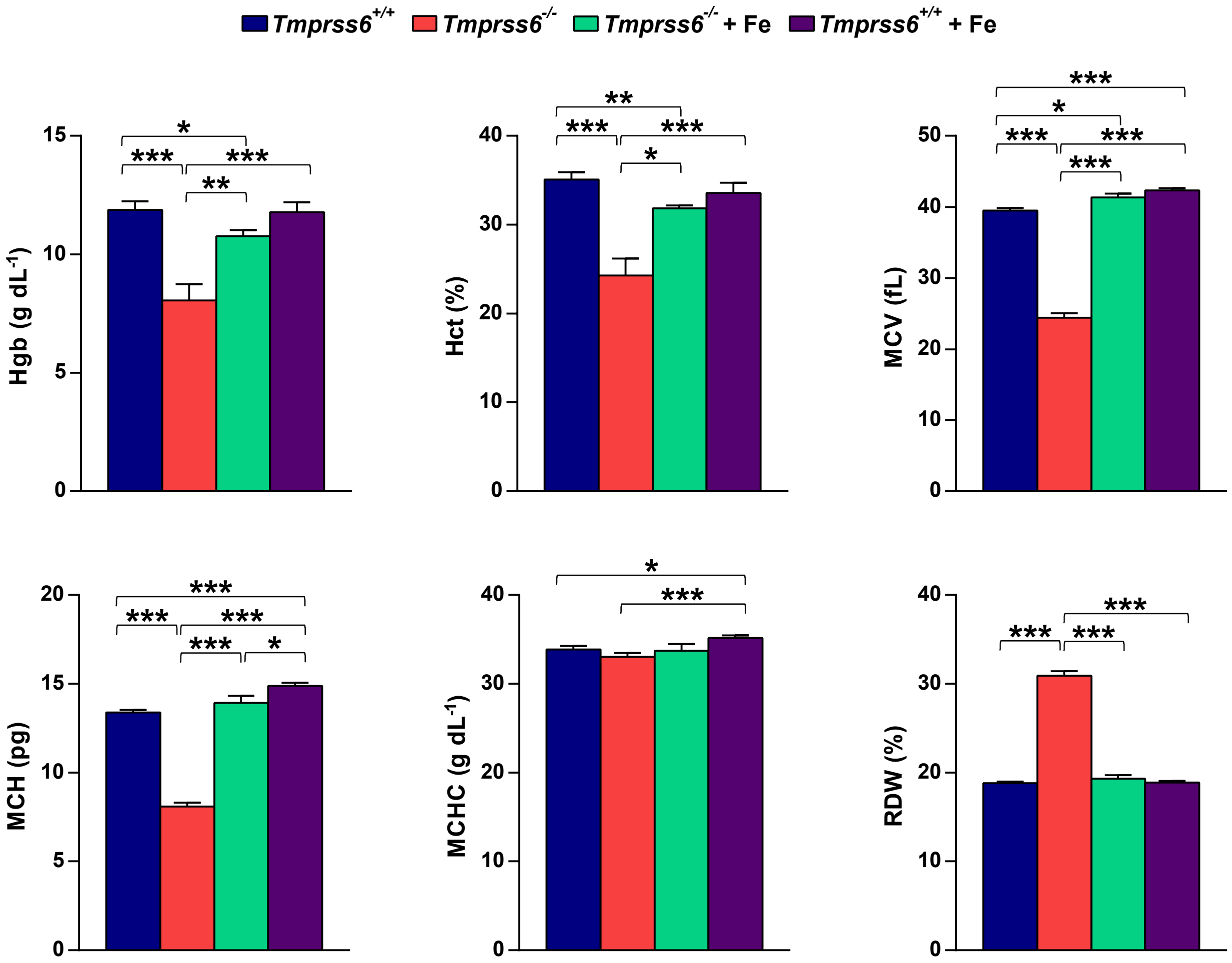

Supplementary Fig. 1. Hematologic parameters of HFD-fed *Tmprss6*$^{+/+}$, *Tmprss6*$^{-/-}$ and both iron-treated *Tmprss6*$^{-/-}$ and *Tmprss6*$^{+/+}$ mice. Complete blood counts were measured from whole blood of *Tmprss6*$^{+/+}$ (n=8), *Tmprss6*$^{-/-}$ (n=7), and both iron-treated *Tmprss6*$^{-/-}$ and *Tmprss6*$^{+/+}$ mice (n=6 and n=9 respectively) fed a HFD for 20 weeks upon overnight fasting. Hgb, hemoglobin; Hct, hematocrit; MCV, mean corpuscular volume; MCH, mean corpuscular hemoglobin; MCHC, mean corpuscular hemoglobin concentration; RDW, red cell distribution width. Data shown are mean ± SEM. *P<0.05, **P<0.01, ***P<0.001, two-tailed Student's *t* test and Mann-Whitney test.

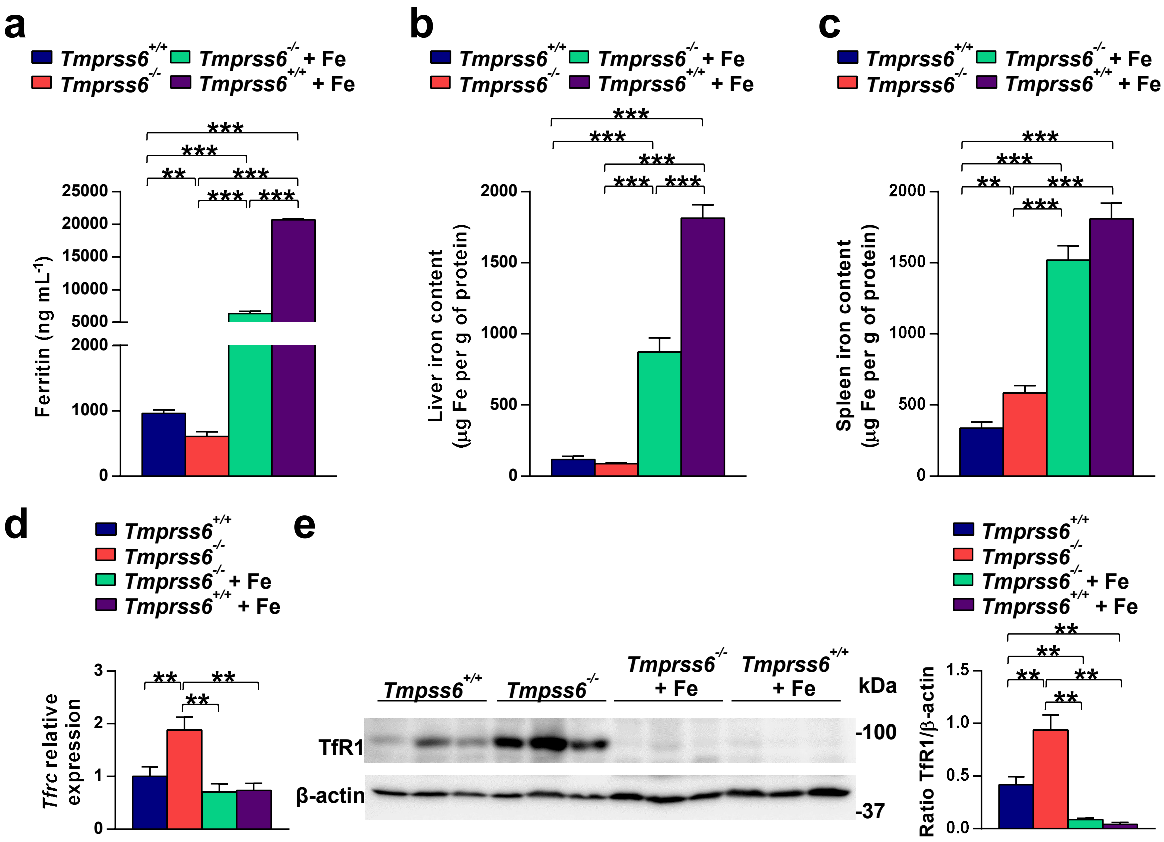

Supplementary Fig. 2. Altered iron homeostasis in *Tmprss6*$^{-/-}$ and both iron-treated *Tmprss6*$^{-/-}$ and *Tmprss6*$^{+/+}$ mice. (**a**) Fasting plasma concentration of ferritin in HFD-fed *Tmprss6*$^{+/+}$ (n=8), *Tmprss6*$^{-/-}$ (n=6), and both iron-treated *Tmprss6*$^{-/-}$ and *Tmprss6*$^{+/+}$ mice (n=7 respectively). (**b**) Liver iron content relative to total protein in HFD-fed *Tmprss6*$^{+/+}$ (n=9), *Tmprss6*$^{-/-}$ (n=8), and both iron-treated *Tmprss6*$^{-/-}$ and *Tmprss6*$^{+/+}$ mice (n=9 and n=8 respectively). (**c**) Spleen iron content relative to total protein in HFD-fed *Tmprss6*$^{+/+}$ (n=6), *Tmprss6*$^{-/-}$ (n=5), and both iron-treated *Tmprss6*$^{-/-}$ and *Tmprss6*$^{+/+}$ mice (n=7 and n=8 respectively). (**d**) Relative gene expression of *Tfrc* in liver samples from HFD-fed *Tmprss6*$^{+/+}$ (n=11), *Tmprss6*$^{-/-}$ (n=9), and both iron-treated *Tmprss6*$^{-/-}$ and *Tmprss6*$^{+/+}$ mice (n=10 and n=8 respectively). (**e**) Western-blot analysis of TfR1 protein expression in liver samples from HFD-fed *Tmprss6*$^{+/+}$ (n=6), *Tmprss6*$^{-/-}$ (n=6), and both iron-treated *Tmprss6*$^{-/-}$ and *Tmprss6*$^{+/+}$ mice (n=6 and n=7 respectively). (left) A representative result showing increased TfR1 protein levels in hypoferremic *Tmprss6*$^{-/-}$ mice compared to wild-types and iron-treated mice. (right) Quantification of TfR1 protein levels relative to loading control β-actin. Data shown are mean ± SEM. *P<0.05, **P<0.01, ***P<0.001, two-tailed Student's t test and Mann-Whitney test.

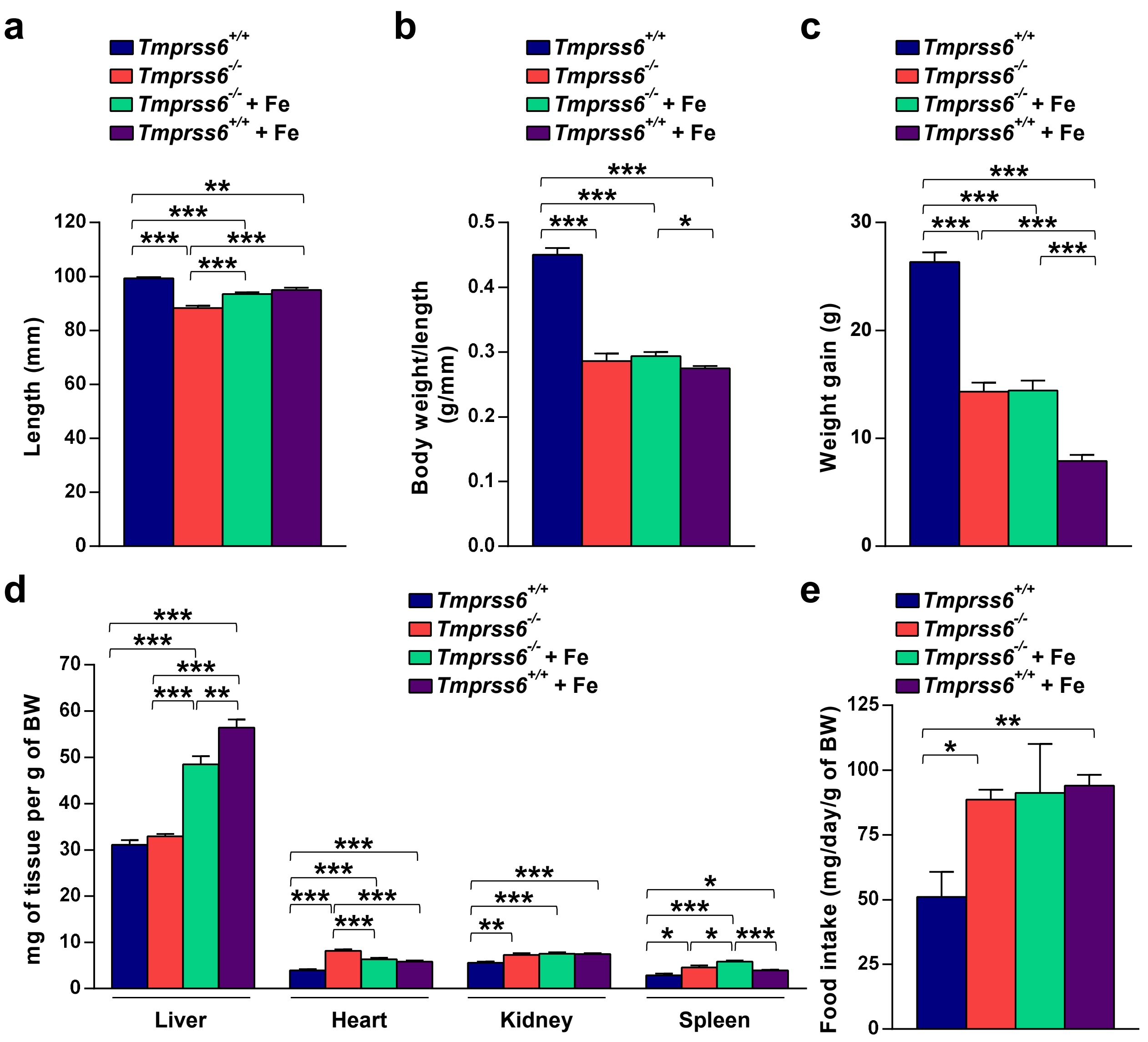

Supplementary Fig. 3. Reduced body weight and increased food intake in *Tmprss6*-deficient mice upon HFD feeding. Length (**a**), body weight relative to length (**b**) and weight gain (**c**) of *Tmprss6*$^{+/+}$ (n=7), *Tmprss6*$^{-/-}$ (n=10), and both iron-treated *Tmprss6*$^{-/-}$ and *Tmprss6*$^{+/+}$ mice (n=11-13 and n=10 respectively) fed a HFD for 20 weeks. (**d**) Organs weight relative to body weight of the same mice. (**e**) Food consumption of *Tmprss6*$^{+/+}$ (n=5), *Tmprss6*$^{-/-}$ (n=5), and both iron-treated *Tmprss6*$^{-/-}$ and *Tmprss6*$^{+/+}$ mice (n=5 and n=6 respectively) fed a HFD. Data shown are mean ± SEM. **P*<0.05, ***P*<0.01, ****P*<0.001, two-tailed Student's *t* test and Mann-Whitney test.

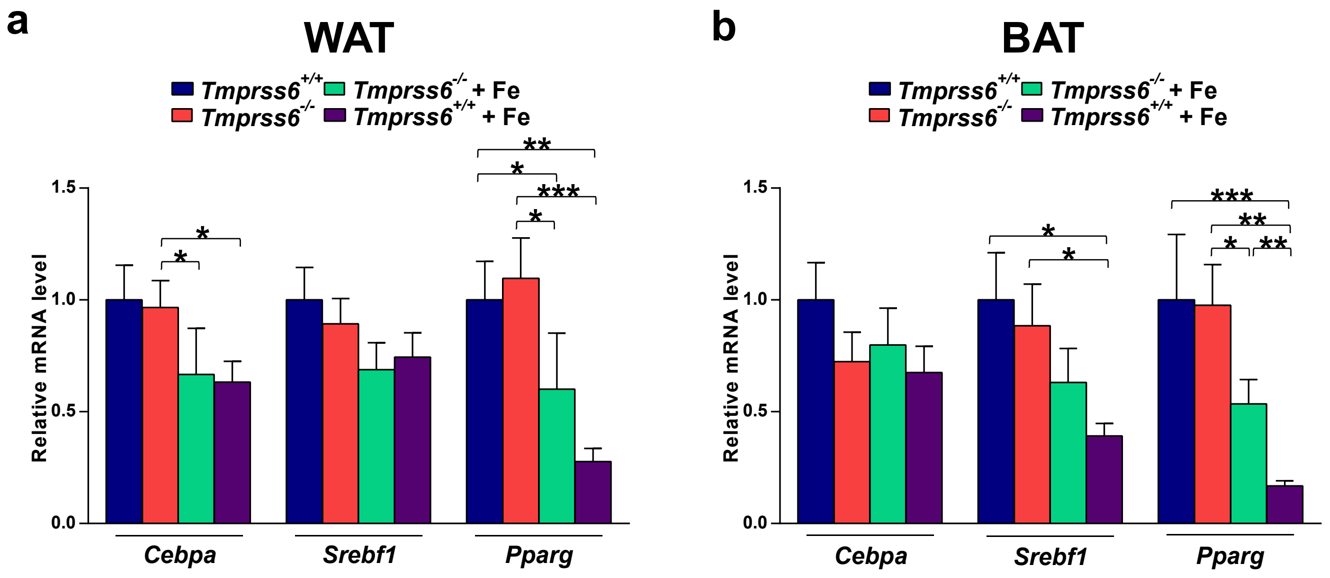

Supplementary Fig. 4. Gene expression analysis of adipocyte differentiation markers in *Tmprss6*-deficient mice fed a high-fat diet. Relative expression levels of genes involved in adipocyte differentiation in WAT (**a**) and BAT (**b**) samples from HFD-fed *Tmprss6*$^{+/+}$ (n=11), *Tmprss6*$^{-/-}$ (n=7-9), and both iron-treated *Tmprss6*$^{-/-}$ and *Tmprss6*$^{+/+}$ mice (n=11 and n=8 respectively). Data shown are mean ± SEM. **P*<0.05, ***P*<0.01, two-tailed Student's *t* test and Mann-Whitney test.

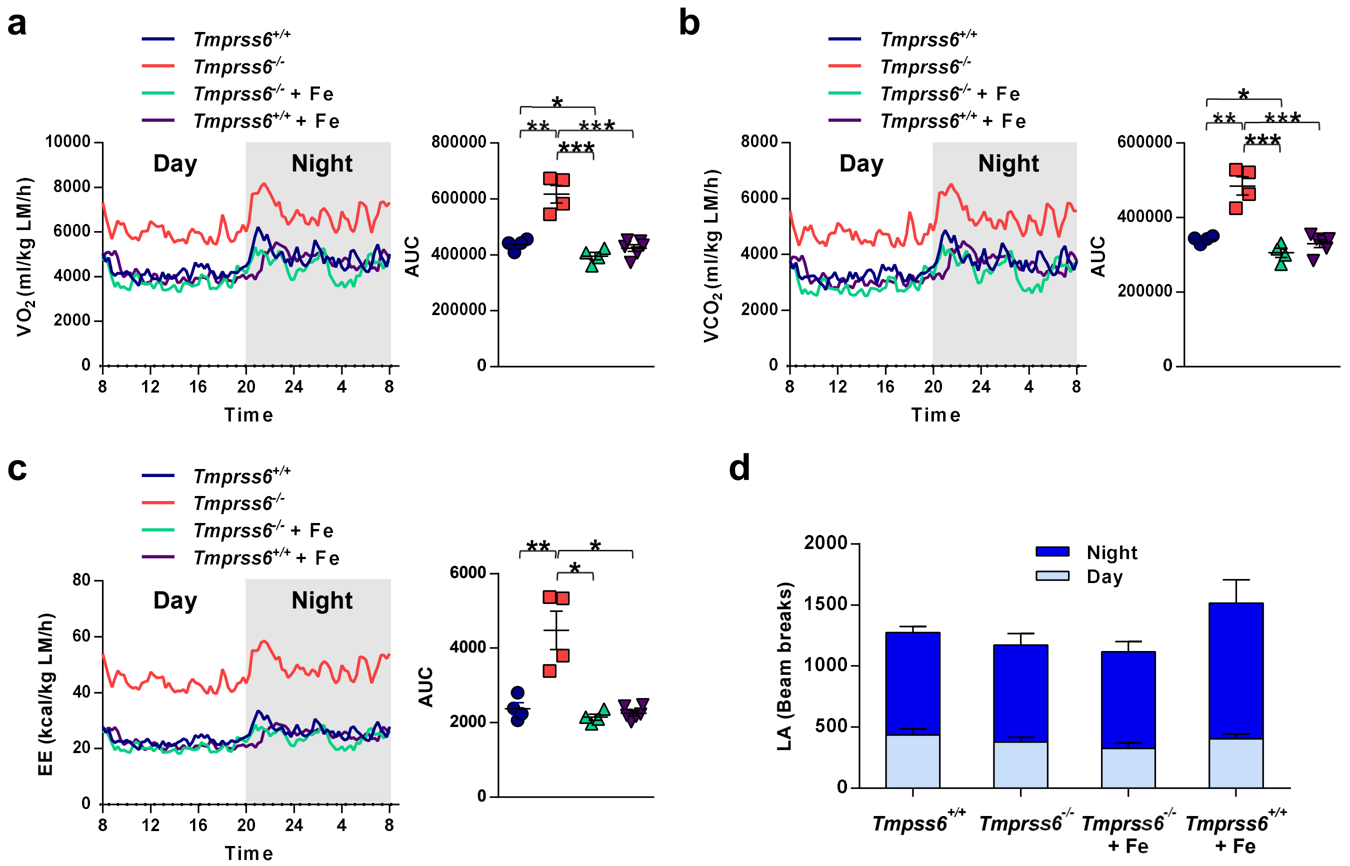

Supplementary Fig. 5. Energy expenditure and locomotor activity of HFD-fed *Tmprss6*-deficient mice. Oxygen consumption rate (VO_2) (**a**), carbon dioxide production (VCO_2) (**b**) and energy expenditure (EE) (**c**) in HFD-fed *Tmprss6*$^{+/+}$ (n=4), *Tmprss6*$^{-/-}$ (n=4), and both iron-treated *Tmprss6*$^{-/-}$ and *Tmprss6*$^{+/+}$ mice (n=4 and n=6 respectively). Data are normalized to lean mass. (**d**) Total ambulatory activity of HFD-fed *Tmprss6*$^{+/+}$ (n=4), *Tmprss6*$^{-/-}$ (n=5), and both iron-treated *Tmprss6*$^{-/-}$ and *Tmprss6*$^{+/+}$ mice (n=5 and n=6 respectively). Data were determined by indirect calorimetry during a 24 h period. AUC, area under the curve. Data shown are mean ± SEM. *P<0.05, **P<0.01, ***P<0.001, two-tailed Student's *t* test.

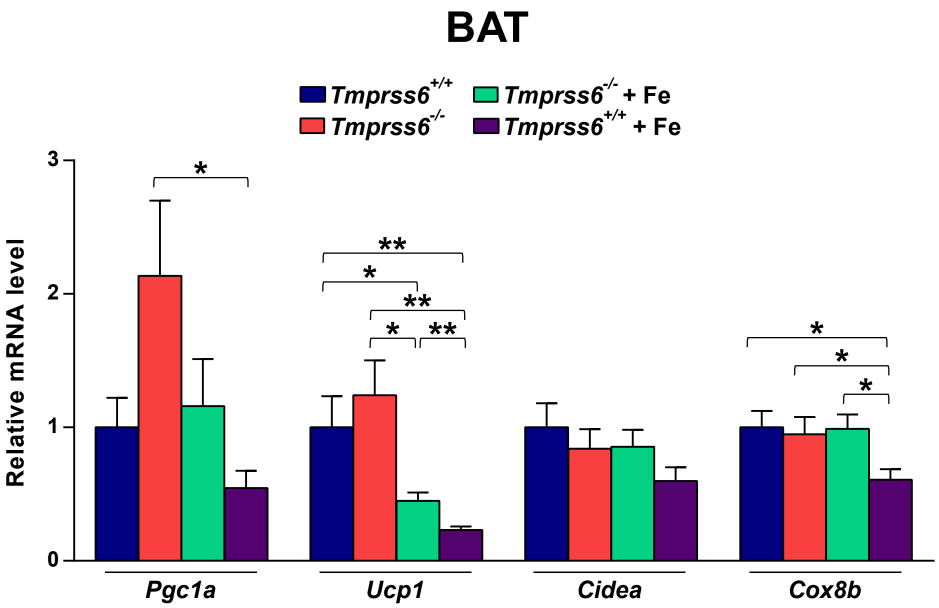

Supplementary Fig. 6. Gene expression analysis of thermogenic genes in *Tmprss6*-deficient mice fed a high-fat diet. Relative expression levels of thermogenic genes in BAT samples of HFD-fed *Tmprss6*$^{+/+}$ (n=11), *Tmprss6*$^{-/-}$ (n=8-9), and both iron-treated *Tmprss6*$^{-/-}$ and *Tmprss6*$^{+/+}$ mice (n=10 and n=8 respectively). Data shown are mean ± SEM. **P*<0.05, ***P*<0.01, two-tailed Student's *t* test.

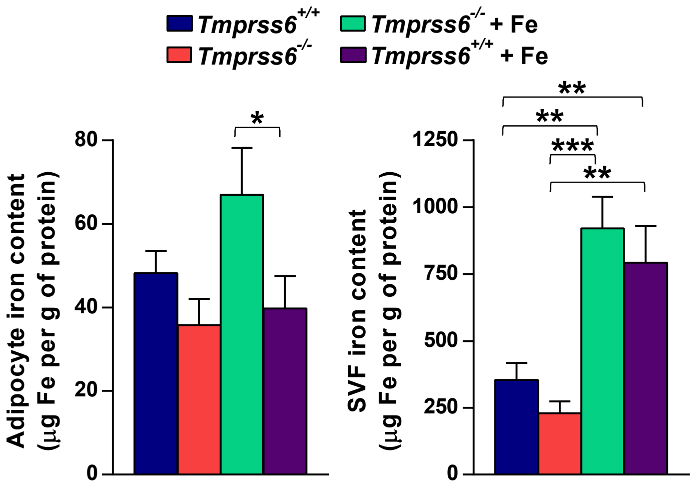

Supplementary Fig. 7. Adipocyte iron content in HFD fed *Tmprss6*-deficient mice. Adipocytes and the stromal vascular fraction (SVF) were isolated from WAT samples of HFD-fed *Tmprss6*$^{+/+}$ (n=5-6), *Tmprss6*$^{-/-}$ (n=5), and both iron-treated *Tmprss6*$^{-/-}$ and *Tmprss6*$^{+/+}$ mice (n=6 and n=8 respectively) and iron levels were determined relative to total protein. Data shown are mean ± SEM. **P*<0.05, ***P*<0.01, ****P*<0.001, two-tailed Student's *t* test and Mann-Whitney test.

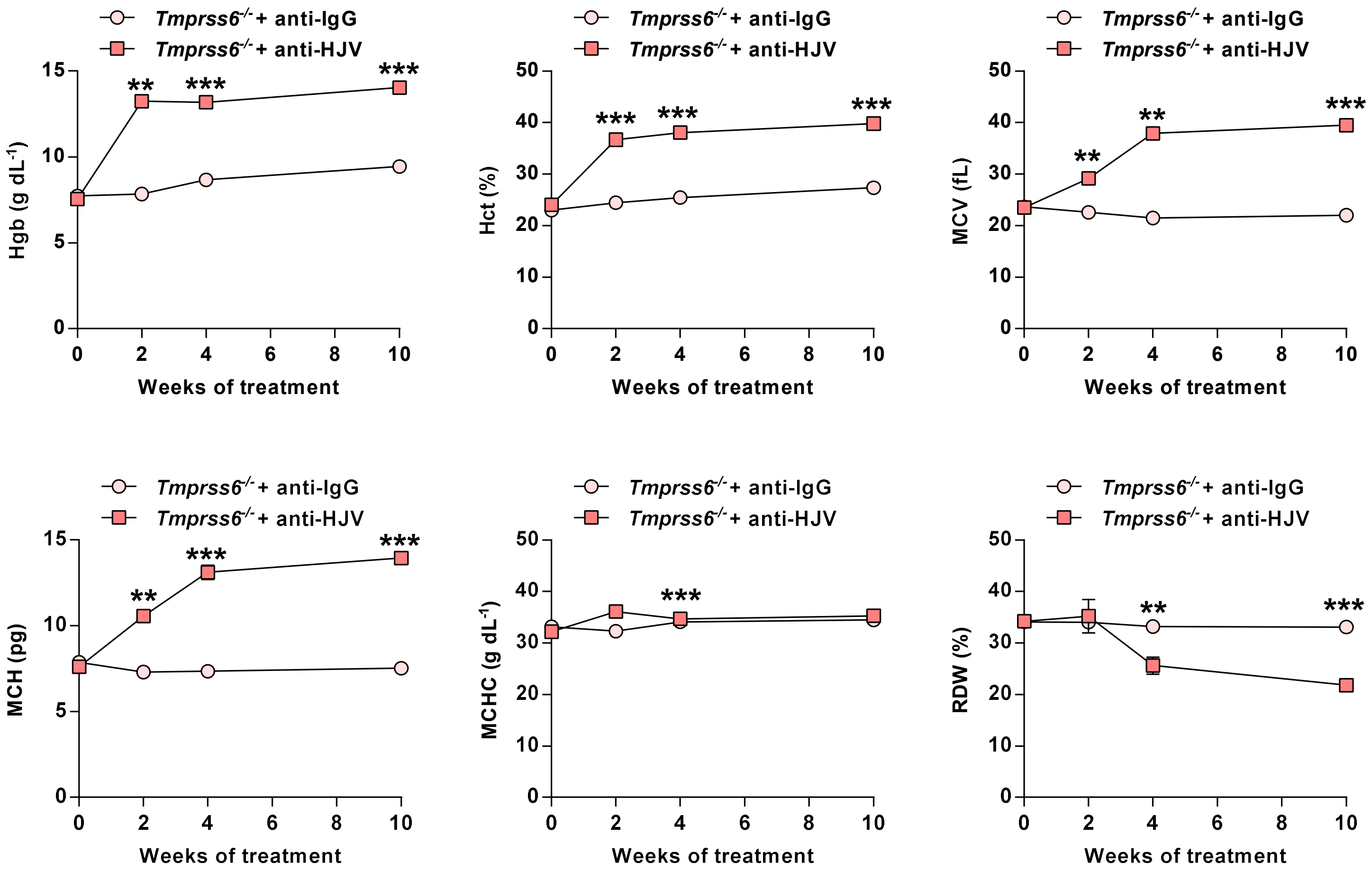

Supplementary Fig. 8. Anti-HJV therapy rescues the hematologic parameters of *Tmprss6*$^{-/-}$ treated mice. Complete blood counts were measured from whole blood of HFD-fed anti-IgG-treated *Tmprss6*$^{-/-}$ (n=3-5) and anti-HJV-treated *Tmprss6*$^{-/-}$ (n=4-8) mice to monitor the efficacy of the treatment over time. Hgb, hemoglobin; Hct, hematocrit; MCV, mean corpuscular volume; MCH, mean corpuscular hemoglobin; MCHC, mean corpuscular hemoglobin concentration; RDW, red cell distribution width. Data shown are mean ± SEM. ***P*<0.01, ****P*<0.001, two-tailed Student's *t* test and Mann-Whitney test.

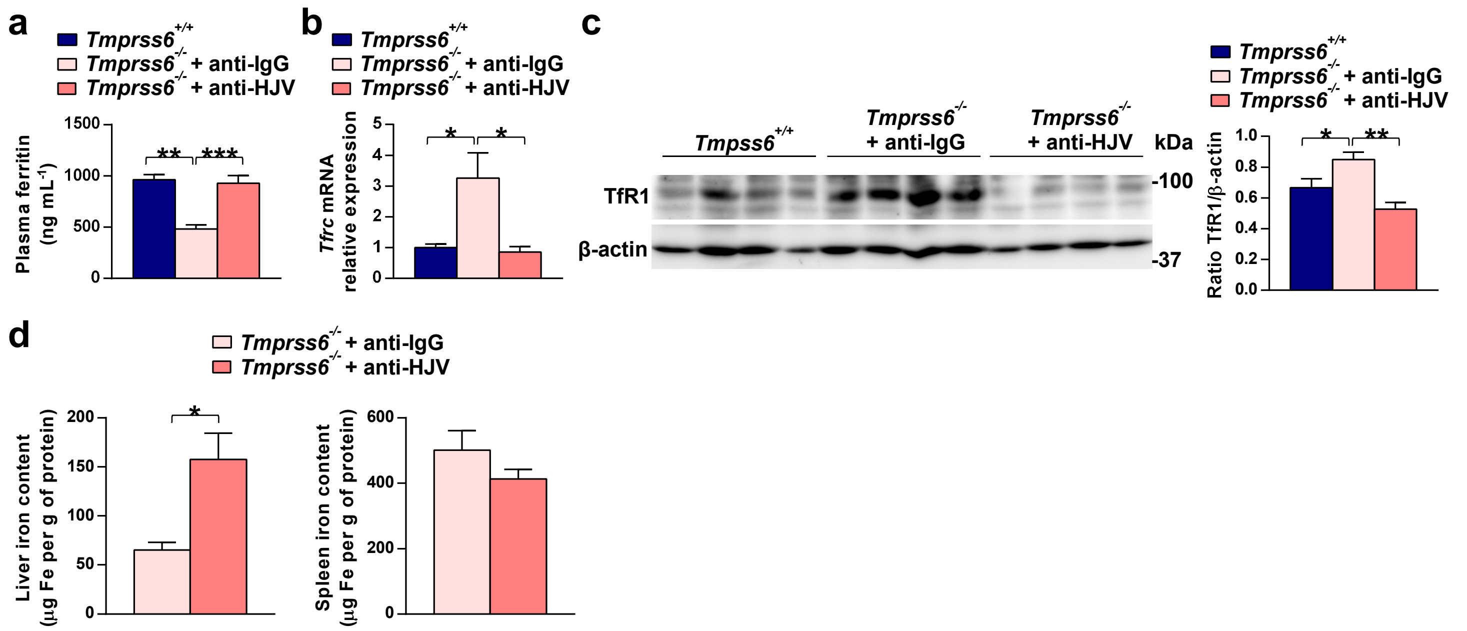

Supplementary Fig. 9. Restored iron homeostasis in *Tmprss6*$^{-/-}$ mice upon anti-HJV therapy. (**a**) Fasting plasma concentration of ferritin in HFD-fed *Tmprss6*$^{+/+}$ (n=8), anti-IgG-treated *Tmprss6*$^{-/-}$ (n=5), and anti-HJV-treated *Tmprss6*$^{-/-}$ (n=8) mice. (**b**) Relative gene expression of *Tfrc* in liver samples from HFD-fed *Tmprss6*$^{+/+}$ (n=7), anti-IgG-treated *Tmprss6*$^{-/-}$ (n=5), and anti-HJV-treated *Tmprss6*$^{-/-}$ (n=8) mice. (**c**) Western-blot analysis of TfR1 protein expression in liver samples from HFD-fed *Tmprss6*$^{+/+}$ (n=4), anti-IgG-treated *Tmprss6*$^{-/-}$ (n=4), and anti-HJV-treated *Tmprss6*$^{-/-}$ (n=5) mice. (left) A representative result showing decreased TfR1 protein levels in *Tmprss6*$^{-/-}$ anti-HJV treated mice compared to *Tmprss6*$^{-/-}$ anti-IgG treated mice. (right) Quantification of TfR1 protein levels relative to loading control β-actin. (**d**) Liver (left) and spleen (right) iron content relative to total protein in HFD-fed anti-IgG-treated *Tmprss6*$^{-/-}$ (n=5), and anti-HJV-treated *Tmprss6*$^{-/-}$ (n=8) mice. Data shown are mean ± SEM. *P<0.05, **P<0.01, ***P<0.001, two-tailed Student's t test.

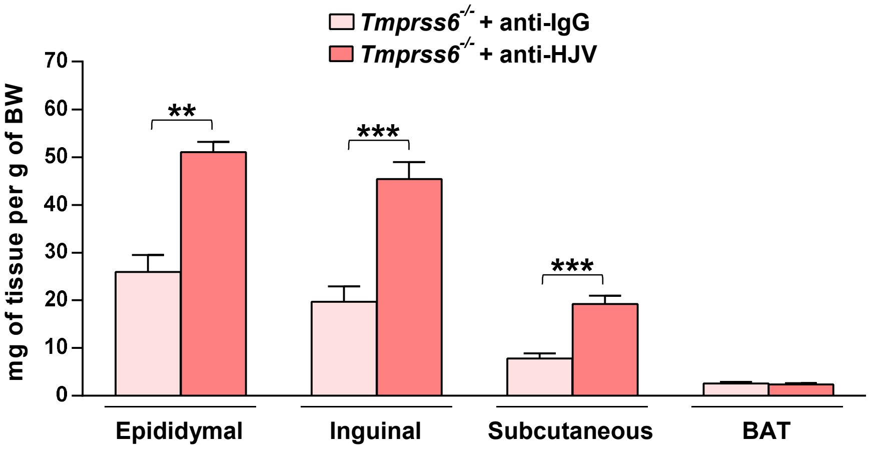

Supplementary Fig. 10. *Tmprss6*$^{-/-}$ mice increase their fat mass upon anti-HJV therapy. White adipose (epididymal, subcutaneous and inguinal) and brown adipose tissue (BAT) masses were determined relative to body weight in anti-IgG-treated *Tmprss6*$^{-/-}$ (n=5), and anti-HJV-treated *Tmprss6*$^{-/-}$ (n=8) mice fed a HFD for 20 weeks. Data shown are mean ± SEM. ***P*<0.01, ****P*<0.001, two-tailed Student's *t* test.

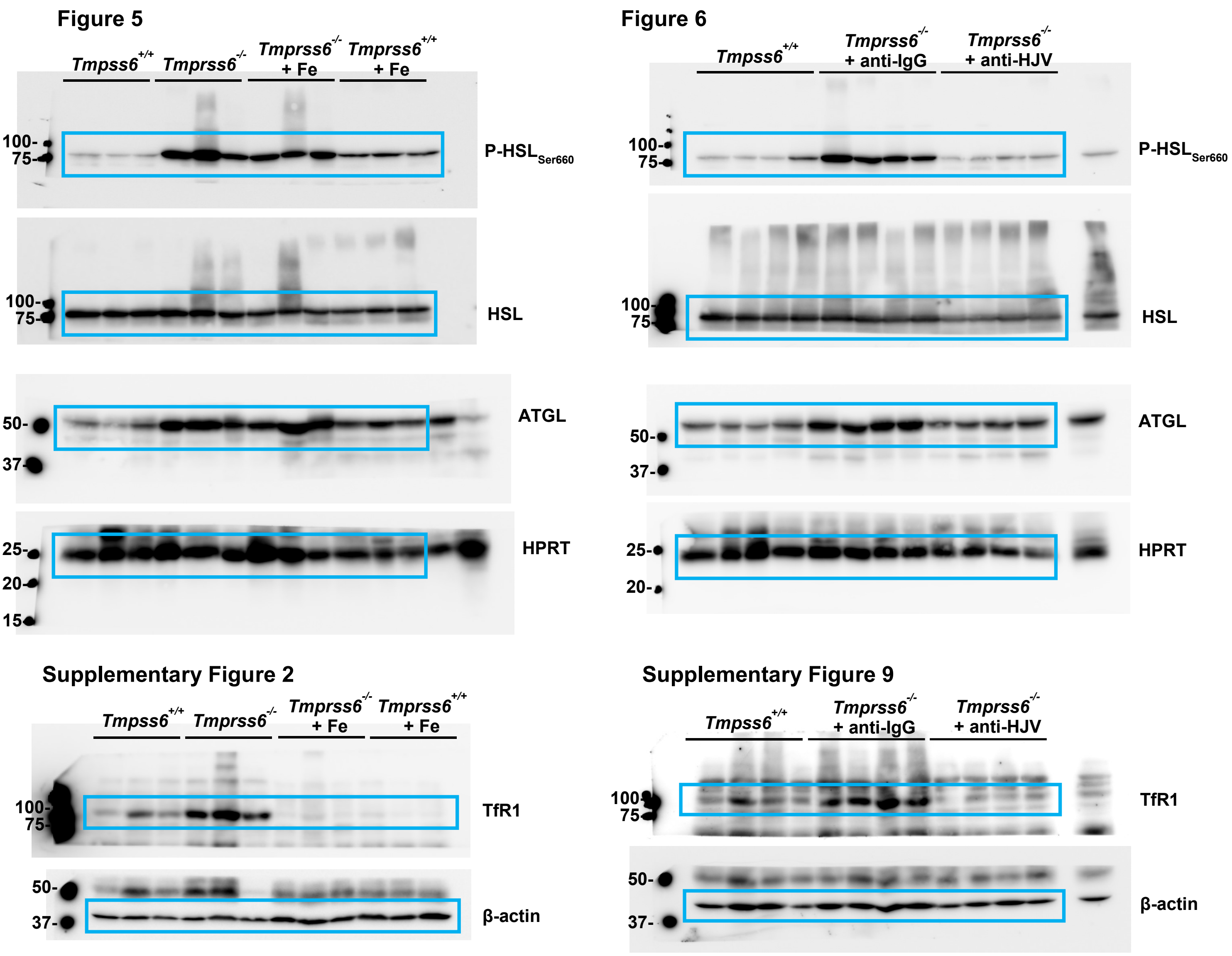

Supplementary Fig. 11. Uncropped images of Western-blots from main and supplementary figures.

Supplementary Table 1. Summary of HFD-induced phenotypes in mouse models of iron imbalance and hepcidin up-regulation.

Mouse model	*Tmprss6*$^{+/+}$	*Tmprss6*$^{-/-}$	*Tmprss6*$^{-/-}$ + Fe	*Tmprss6*$^{+/+}$ + Fe
**Iron status**	Basal	Hypoferremia	Iron overload	Iron overload
**Hepcidin levels**	Basal	Up-regulated ↑	Up-regulated ↑↑	Up-regulated ↑↑
**Fat mass**	~ 40 %	~ 26 %	~ 13 %	~ 15 %
**Leptin levels**	Basal	Low	Low	Low
**Hepatic steatosis**	Yes	No	No	No
**Glucose tolerance**	Low	Increased	Increased	Increased
**Insulin sensitivity**	Low	Increased	Moderated	Moderated
**Energy expenditure**	Basal	Increased	Basal	Basal
**Fat lipolysis**	Basal	Increased	Increased	Increased

Supplementary Table 2. Primer sequences used for real-time quantitative PCR

Gene	Forward primer (5’-3’)	Reverse primer (5’-3’)
Acaca	CTGTATGAGAAAGGCTATGTG	AACCTGTCTGAAGAGGTTAG
Actb	CTGAGGAGCACCCTGTGCT	GTTGAAGGTCTCAAACATGATCTG
Adrb3	CCAAGCTACACGATGCCATGT	GAGCTCAGCAGCTCCTTCCT
Cebpa	CAAGAACAGCAACGAGTACCG	GTCACTCGTCAACTCCAGCAC
Cidea	TGACATTCATGGGATTGCAGAC	GGCCAGTTGTGATGACTAAGAC
Cox8b	GCGAAGTTCACAGTGGTTCC	GAACCATGAAGCCAACGACT
Cpt1a	GGGAGGAATACATCTACCTG	GAAGACGAATAGGTTTGAG
Fasn	GATTCAGGGAGTGGATATTG	CATTCAGAATCGTGGCATAG
Fsp27	ATGGACTACGCCATGAAGTCT	CGGTGCTAACACGACAGGG
Fbp1	AAGTACTGATGAGCCTTCTG	GCTCACCATAATGAATTCTCC
G6pc	TTCAAGTGGATTCTGTTTGG	AGATAGCAAGAGTAGAAGTGAC
Gck	TGTACGAAAAGATCATTGGC	TCAGGATGTTAAGGATCTGC
Lipe	CAT GGCTCAACTCCTTCCTG	CTG TGCCCAGTAAGCCCTCA
Pck1	AATATGACAACTGTTGGCTG	AATGCTTTCTCAAAGTCCTC
Pfk1	AAGAGACTGATTTTGAGCAC	CTCAGAAACCCTTGTCTATG
Pgc1a	TCCTCTTCAAGATCCTGTTA	CACATACAAGGGAGAATTGC
Pklr	GTGAAGAAGTTTGATGAGATCC	CAAGAAAACCTTCTCTGCTG
Pnpla2	CAACCTTCGCAATCTCTAC	TTCAGTAGGCCATTCCTC
Pparg	TCGCTGATGCACTGCCTATG	GAGAGGTCCACAGAGCTGATT
Rn18s	GTAACCCGTTGAACCCCATT	CCATCCAATCGGTAGTAGCG
Scd1	GTGGGGTAATTATTTGTGACC	TTTTTCCCAGACAGTACAAC
Srebf1	GATGTGCGAACTGGACACAG	CATAGGGGGCGTCAAACAG
Tfrc	CCCATGACGTTGAATTGAACCT	GTAGTCTCCACGAGCGGAATA
Ucp1	CTTTTTCAAAGGGTTTGTGG	CTTATGTGGTACAATCCACTG
